# DFT computational insight into the catalytic dehydrogenation of formic acid for H_2_ production over Cr-doped C_20_ fullerene: Cr–H active center and high selectivity mechanism

**DOI:** 10.1039/d6ra05021b

**Published:** 2026-08-07

**Authors:** Jihong Xu, Aili Feng, Shiling Yuan, Hongjun Zhou, Dongju Zhang

**Affiliations:** a School of Chemical Engineering, Carbon Neutrality Research Institute, Shandong Key Laboratory of Green Electricity & Hydrogen Science and Technology, Shandong Institute of Petroleum and Chemical Technology Dongying 257061 P. R. China shilingyuan@sdu.edu.cn zhouhongjun@cup.edu.cn +86-0531-88564464 +86-0531-88365833; b Key Lab of Colloid and Interface Chemistry, Ministry of Education, School of Chemistry and Chemical Engineering, Shandong University Jinan 250100 P. R. China zhangdj@sdu.edu.cn

## Abstract

The dehydrogenation of formic acid (HCOOH) to H_2_ offers a sustainable energy conversion route, yet developing efficient non-noble metal catalysts remains a critical challenge. Herein, density functional theory (DFT) calculations were performed to systematically investigate the catalytic performance of Cr-doped C_20_ fullerene (C_19_Cr) for HCOOH dehydrogenation. Our results show that the reaction follows an O–H bond-prior activation mechanism: the first HCOOH molecule dissociates to form a Cr–H hydride, which serves as the catalytically active center, and subsequent reaction with a second HCOOH molecule yields H_2_. The formation of the Cr–H hydride is identified as the rate-determining step, featuring a moderate barrier of 21.2 kcal mol^−1^, confirming kinetic feasibility under mild conditions. Notably, the undesired dehydration pathway (to H_2_O and CO) is kinetically and thermodynamically unfavorable compared to the target dehydrogenation pathway (to H_2_ and CO_2_), endowing C_19_Cr with high H_2_ selectivity. This work highlights the potential of Cr-doped fullerenes as non-noble metal catalysts for HCOOH dehydrogenation and provides valuable theoretical insights for the rational design of efficient H_2_ storage/utilization catalysts.

## Abstract 2

Cr-doped C_20_ fullerene exhibits efficient and selective catalytic performance for formic acid dehydrogenation to H_2_, with Cr–H hydride as the active center that kinetically suppresses the dehydration side reaction, as revealed by DFT calculations.

## Graphical abstract

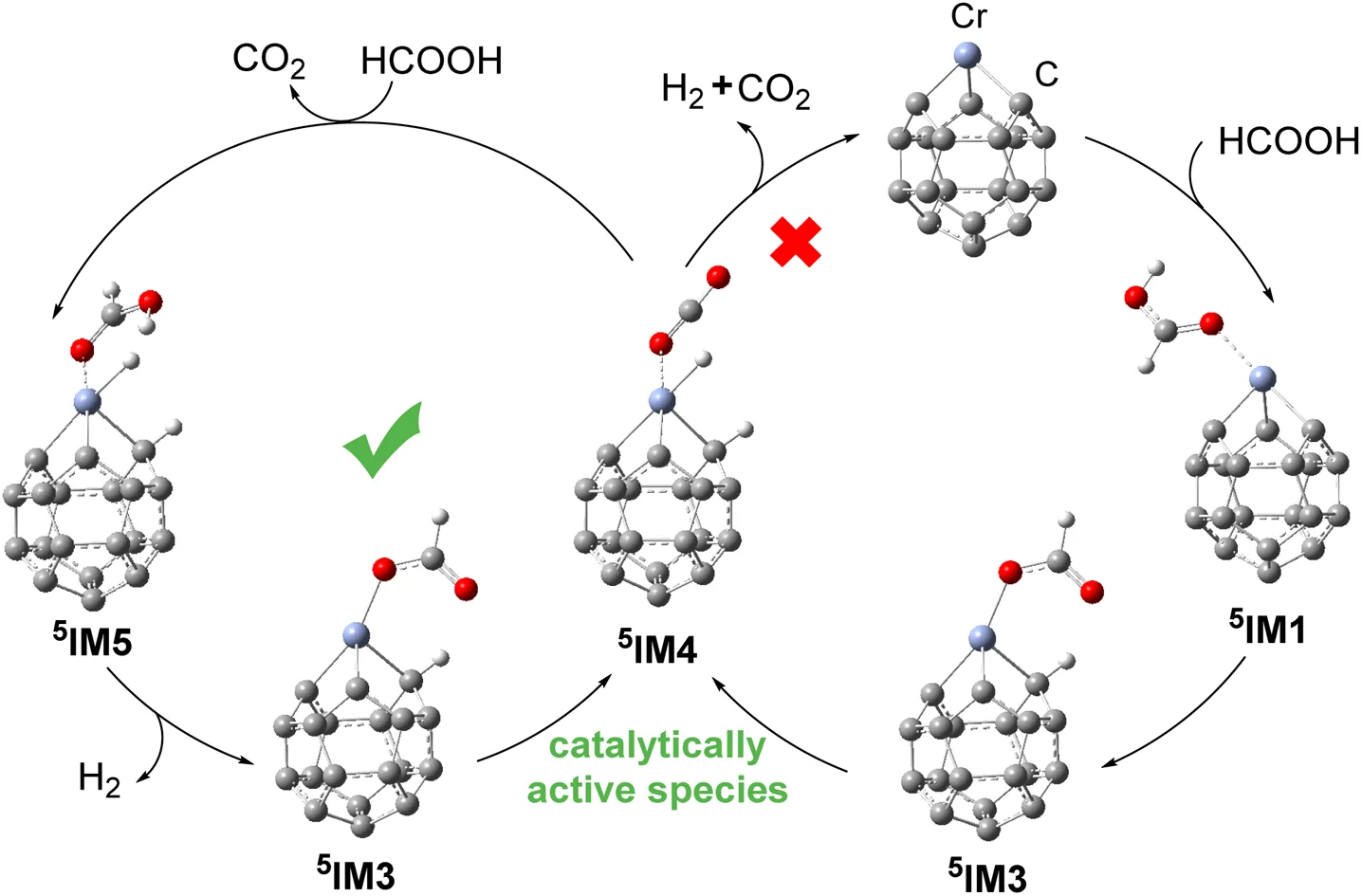

## 1 Introduction

**Scheme 1:**
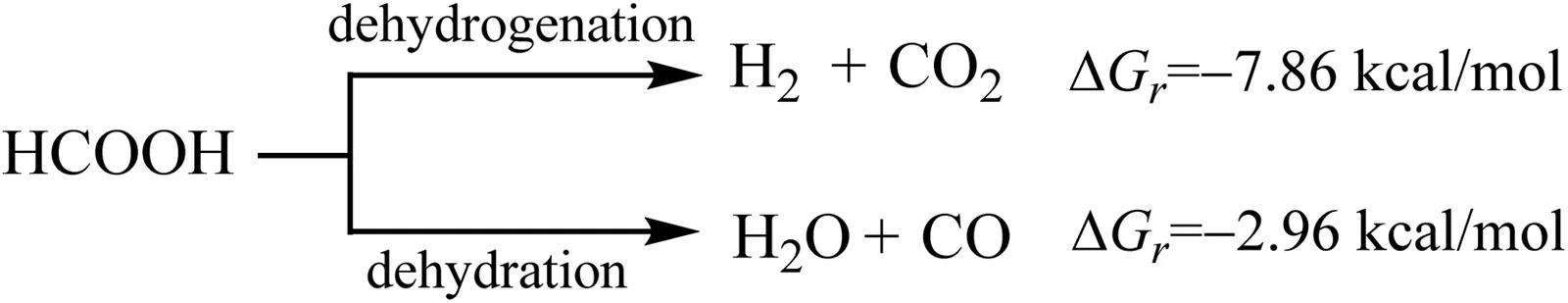
Two decomposition pathways of formic acid (HCOOH) with corresponding reaction energies.

Hydrogen energy has gradually emerged as a promising sustainable energy source for effectively resolving fossil fuels scarcity and reducing carbon emissions.^1–3^ However, the practical application of hydrogen (H_2_) is hindered by its high flammability and low volumetric density under standard conditions, making safe storage and long-distance transportation critical challenges.^4–6^ Liquid organic hydrogen carriers (LOHCs), including methanol (CH_3_OH), formaldehyde (HCHO), cyclohexane (C_6_H_12_), and formic acid (HCOOH), offer a viable solution by enabling efficient hydrogen storage and release in liquid form.^7^ Among these LOHCs, HCOOH stands out due to its high hydrogen storage capacity (4.4 wt% and 53 g H_2_ per L), low toxicity and environmental benignity.^8–10^ Notably, HCOOH decomposition proceeds *via* two competing pathways: dehydrogenation (target pathway yielding H_2_ and CO_2_) and dehydration (undesired pathway producing H_2_O and CO).^11^ As shown in Scheme 1, both the dehydrogenation and dehydration pathways are thermodynamically favorable (Δ*G* = −7.89 and −2.96 kcal mol^−1^, respectively),^12^ with only a modest difference in their reaction Gibbs free energies. Therefore, the product selectivity is expected to be governed primarily by kinetic factors rather than thermodynamics.

The key to unlocking the potential of HCOOH as a hydrogen carrier lies in the identifying highly active and selective catalysts. Consequently, the rational design of catalysts for efficient H_2_ production from HCOOH has garnered widespread attention. Over the past two decades, numerous experimental and theoretical studies have been dedicated to investigating HCOOH dehydrogenation using various catalyst systems.^13–15^ Traditionally, transition metals such as Pd, Pt, and Au have been extensively employed as active components for this reaction.^16–18^ Despite their exceptional catalytic performance, noble-metal catalysts are hampered by scarcity and high cost, which severely limit their large-scale industrial application. In response, substantial research efforts have shifted toward the development of non-noble-metal catalysts^19–21^ as sustainable alternatives for HCOOH dehydrogenation.

Carbon-based nanostructures doped or decorated with transition metals have emerged as a promising class of catalysts, integrating the high surface area, tunable electronic properties, and chemical stability of carbon frameworks with the catalytic functionality of transition metals. Recently, non-noble-metal-embedded graphene and fullerenes-based materials have garnered extensive attention as catalyst for HCOOH dehydrogenation because of their low cost, high catalytic activity, and structural flexibility, although their pristine counterparts generally exhibit negligible catalytic activity toward formic acid dehydrogenation. In 2021, Anafcheh *et al.*^22^ designed an aluminum-ligated pincer fullerene catalyst *via* density functional calculations (DFT) calculations, achieving efficient HCOOH dehydrogenation to generate H_2_ through metal–ligand cooperation. In 2022, Karaman *et al.*^23^ reported the HCOOH decomposition pathways on nickel (Ni) and copper (Cu)-embedded graphene surfaces, revealing distinct bond cleavage energy barriers, differing dominant reactive intermediates and final products on the two substrates. Both the studies offer valuable guidance for developing low-cost non-noble metal catalysts to replace noble metals for HCOOH dehydrogenation reactions.

Among transition metals, Cr has attracted considerable attention as a promising dopant for carbon-based catalysts because its partially filled 3d orbitals can effectively modulate the electronic structure of carbon nanomaterials and generate catalytically active sites. Previous theoretical studies have demonstrated that Cr doping can significantly alter the physicochemical properties of carbon nanostructures. For example, density functional theory (DFT) calculations on transition-metal-doped C_20_ fullerenes (Sc, Ti, V, Cr, Mn, Fe, Co, Ni, Cu, and Zn) show that the incorporation of Cr, together with Sc, Ti, V, Mn and Fe, are thermodynamically favorable, whereas Co, Ni, Cu, and Zn doping are endothermic.^24^ In addition, Cr-doped graphene has been reported to exhibit superior adsorption capacities toward CO and CO_2_ compared with other transition-metal-doped graphene systems.^25^ Cr-doped fullerenes have also been proposed as promising catalytic materials based on their favorable adsorption properties toward adenine.^26^ These findings suggest that Cr is an attractive dopant for tuning the catalytic properties of carbon nanomaterials. However, to the best of our knowledge, the investigations into the adsorption and catalytic performance of HCOOH over Cr-doped C_20_ fullerenes have not yet to be reported.

In the present study, a substitutionally Cr-doped C_20_ fullerene (C_19_Cr) was selected as a representative model to investigate the catalytic properties of Cr-doped fullerenes. DFT calculations were employed to systematically explore the dehydrogenation and dehydration of HCOOH mechanism on this model system. The reaction pathways, corresponding energy profiles, and geometrical structures of key intermediates were analyzed in detail to elucidate the underlying catalytic mechanism. The insights gained from this work may provide guidance for the rational design of efficient fullerene-based catalysts for HCOOH dehydrogenation toward sustainable hydrogen energy.

## 2 Computational methods

All DFT calculations were carried out with the Gaussian16 program package.^27^ Geometry optimizations of all stationary points were performed in the gas phase using the M06 functional^28^ combined with a mixed basis set: the SDD basis set^29,30^ for the Cr atom and 6-311G(d,p) basis set^31^ for C, H and O atoms. The M06 functional was selected because it has been widely validated for transition-metal-containing systems and provides reliable descriptions of thermochemistry, reaction barriers, and noncovalent interactions.^32–34^ The mixed basis set provides a good balance between computational cost and accuracy. The SDD basis set incorporates relativistic effective core potentials, making it suitable for describing the Cr center, while the 6-311G(d,p) basis set with polarization functions provides reliable geometries and energetics for C, H, and O atoms. This basis set combination has been widely adopted and validated in previous DFT studies of transition-metal-catalyzed reaction mechanisms.^35,36^ Vibrational frequency analyses were conducted at the same level of theory to characterize the optimized structures, with local minima exhibiting no imaginary frequencies and transition states exhibiting a single imaginary frequency. Intrinsic reaction coordinate (IRC)^37,38^ calculations were subsequently performed to ensure that each transition state correctly connects the corresponding reactants and products. Wiberg bond order analyses^39^ were carried out to evaluate bonding characteristics and atomic orbital populations. The singlet state of C_19_Cr reported in this work was calculated using a closed-shell electronic configuration. All reported Gibbs free energies were calculated at 298.15 K and 1 atm.

## 3 Results and discussion

### 3.1 Ground-state structures of C_19_Cr

The C_19_Cr model was constructed by substituting one carbon atom in the C_20_ fullerene cage with a chromium atom. Pristine C_20_ adopts an ideal cage structure with *I*_h_ symmetry, in which all twenty carbon atoms are symmetry-equivalent. Consequently, substitution at any carbon atom site leads to an identical structure, and thus only a single unique C_19_Cr configuration exists for the present system.

Spin-state effects are known to play an essential role in transition-metal-based catalysis, as different spin configurations can give rise to distinct electronic structures and reaction pathways.^40,41^ Given that the Cr atom possesses six valence electrons in its outer shell, the electronic ground state of the C_19_Cr system was examined by optimizing the singlet (^1^C_19_Cr), triplet (^3^C_19_Cr), quintet (^5^C_19_Cr) and septet (^7^C_19_Cr) spin states and comparing their relative stabilities. In the present calculations, the quintet was identified as the ground state of the C_19_Cr cluster. Although the triplet lies only 3.3 kcal mol^−1^ higher in Gibbs free energy, the singlet and septet states are significantly less stable, with much higher relative free energies of 44.4 and 24.5 kcal mol^−1^, respectively. Such large energy separations render the singlet and septet states energetically inaccessible under typical thermal catalytic conditions. Accordingly, the catalytic HCOOH dehydrogenation pathways were explored only on the quintet and triplet potential energy surfaces. The calculated results on the quintet potential energy surface are shown in the main text and that on the triplet potential energy surface are given in the SI. Our calculated results are consistent with the previous theoretical study by Zhang *et al.*,^42^ who reported that the quintet state of C_19_Cr is 0.03 eV lower in energy than the triplet state. Nevertheless, the small energy difference suggests that the triplet state may also be accessible and should therefore be considered in the investigation of the catalytic reaction pathways.

**Fig. 1:**
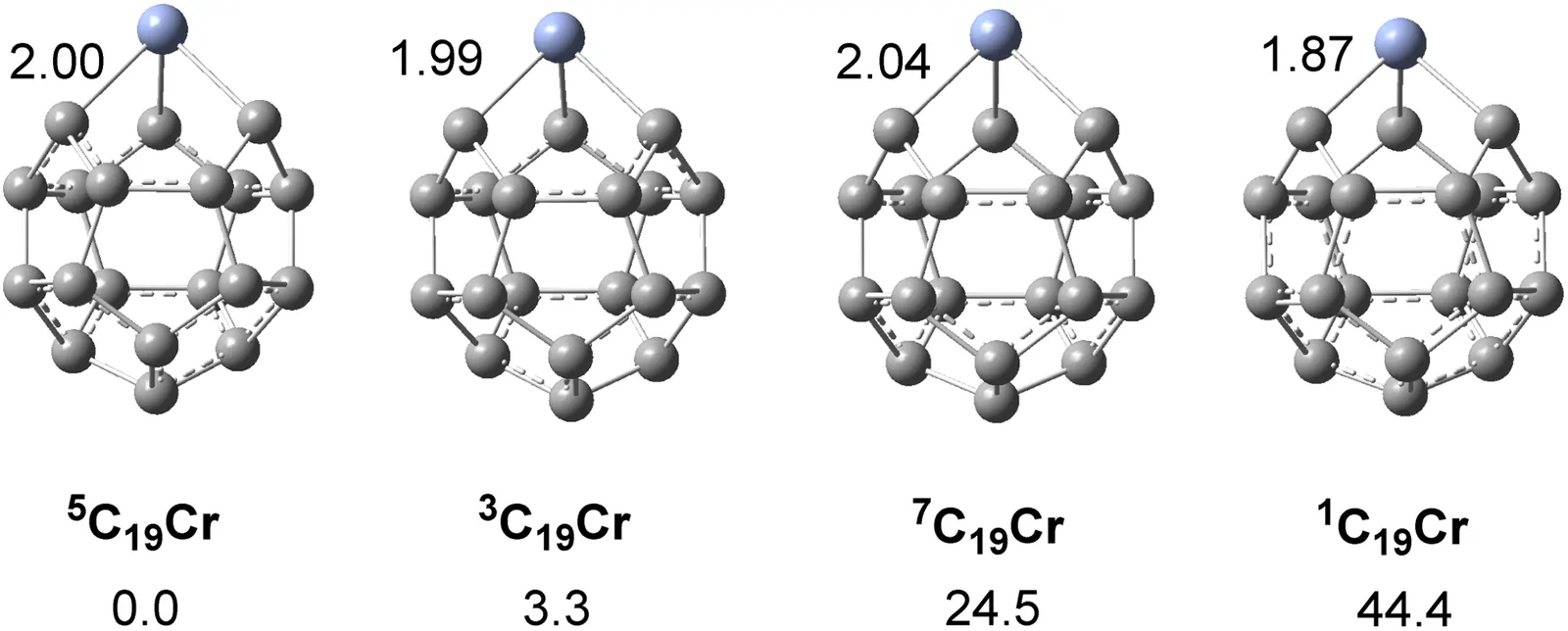
Optimized structures of quintet, triplet, septet, and singlet states of C_19_Cr with calculated Cr–C distances (in Å) and relative energies (in kcal mol^−1^).

The optimized geometries of ^5^C_19_Cr, ^3^C_19_Cr, ^7^C_19_Cr, and ^1^C_19_Cr are shown in Fig. 1. The fullerene framework in all four structures remains essentially intact after substitution, although slight local distortions occur near the dopant site. In these structures, the embedded Cr atom is coordinated to three adjacent carbon atoms with average Cr–C bond lengths of approximately 2.00 Å in ^5^C_19_Cr, 1.99 Å in ^3^C_19_Cr, 2.04 Å in ^7^C_19_Cr, and 1.87 Å in ^1^C_19_Cr. These structural characteristics indicate that the Cr atom can be stably incorporated into the C_20_ fullerene cage, providing a well-defined coordination environment for subsequent adsorption and reaction of HCOOH.

The stability of the ground state C_19_Cr cluster was further evaluated by calculating its formation energy with respect to isolated C and Cr atoms, considering that metal-doped carbon cages are typically formed through the assembly of gaseous carbon and metal atoms under arc-discharge conditions. The calculated formation energy is −5.5 eV atom^−1^, which is only 0.3 eV atom^−1^ less negative than that of pristine C_20_, indicating that Cr doping causes only a slight reduction in the thermodynamic stability. Therefore, the C_19_Cr cluster remains thermodynamically stable and is expected to be experimentally accessible under the non-equilibrium synthesis conditions.

For the decomposition of formic acid, two competing reaction pathways are generally possible. One pathway corresponds to dehydrogenation, producing H_2_ and CO_2_, while the other involves dehydration, leading to the formation of CO and H_2_O. From the perspective of sustainable energy utilization, the dehydrogenation pathway is highly desirable, as it enables the generation of clean hydrogen without CO contamination.

Therefore, the development of suitable catalysts that can selectively promote the dehydrogenation of HCOOH while suppressing the competing dehydration pathway is of significant interest. In this context, both reaction channels were examined in the present study to evaluate whether the C_19_Cr catalyst can achieve the desired activity and selectivity toward HCOOH dehydrogenation.

### 3.2 Dehydrogenation of HCOOH to H_2_ and CO_2_

**Fig. 2:**
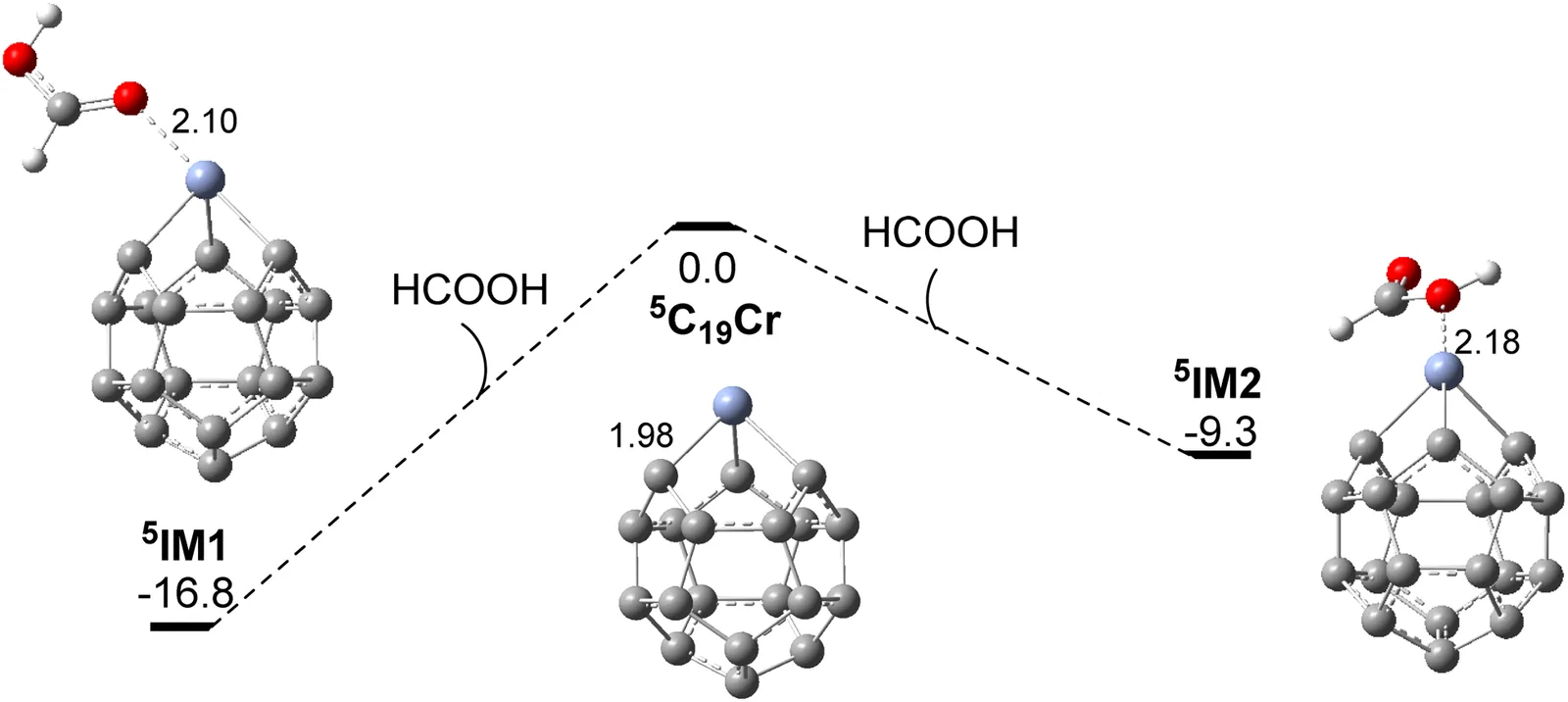
Calculated Gibbs energy profile for the binding of HCOOH on ^5^C_19_Cr. Selected bond distances are given in Å.

The binding of reactants to the catalyst represents the first step of the dehydrogenation of HCOOH. Therefore, the interaction between HCOOH and ^5^C_19_Cr was first examined. As shown in Fig. 2, two possible coordination modes were considered. Coordination of the carbonyl oxygen of HCOOH to the Cr center leads to the formation of ^5^IM1, which is exothermic by 16.8 kcal mol^−1^. This binding mode is 7.5 kcal mol^−1^ more favorable than the alternative configuration in which the hydroxyl oxygen coordinates to the Cr center to form ^5^IM2.

From a mechanistic perspective, two possible pathways can be envisaged for the dehydrogenation of HCOOH. One pathway involves initial cleavage of the hydroxyl O–H bond, followed by activation of the formyl C–H bond. The alternative pathway proceeds *via* initial C–H bond activation, followed by O–H bond cleavage. These two mechanistic routes are discussed separately in the following sections.

#### 3.2.1 O–H bond-first activation mechanism

**Fig. 3:**
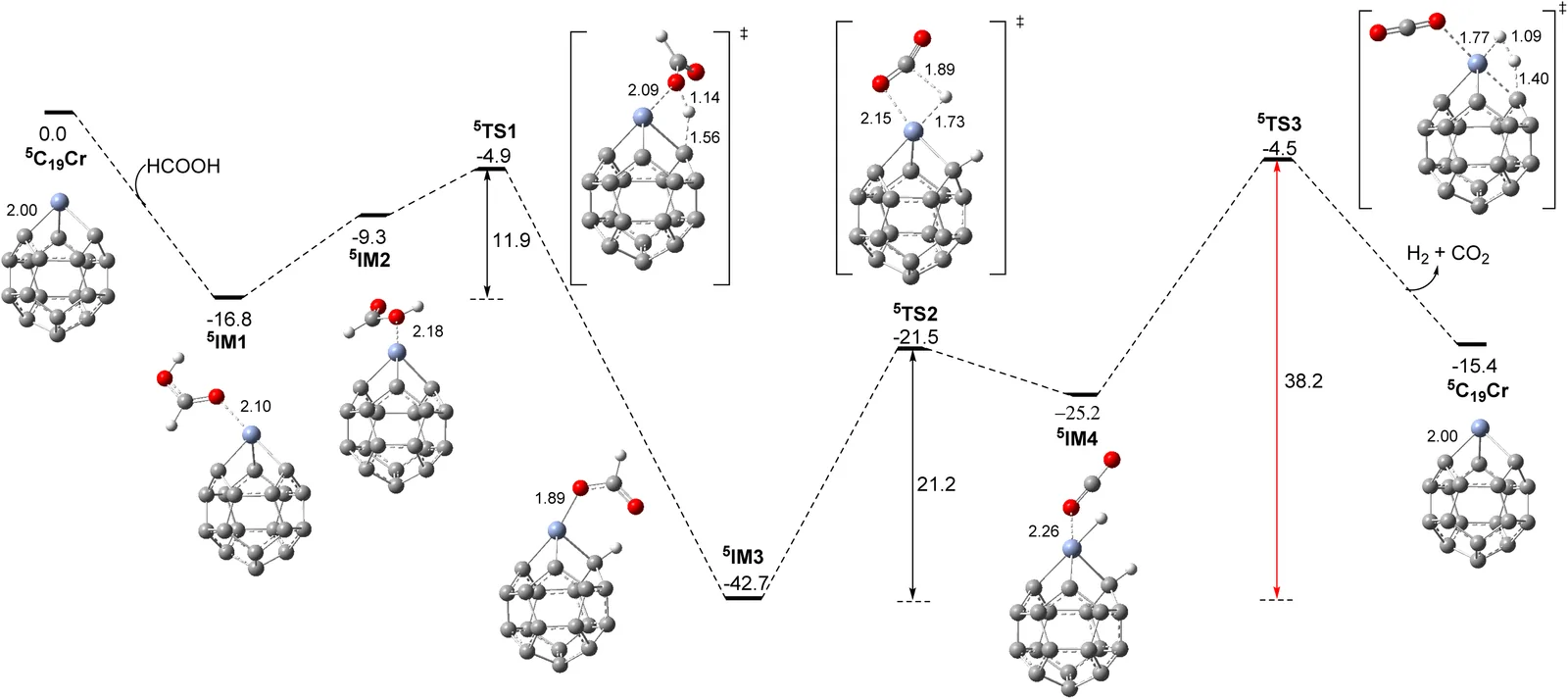
Calculated Gibbs energy profile and optimized structures for the dehydrogenation of HCOOH on ^5^C_19_Cr *via* the O–H bond-first activation mechanism. Relative energies are given in kcal mol^−1^ and selected bond lengths are in Å.

As shown in Fig. 3, ^5^IM1 isomerizes into ^5^IM2, which has been identified as the precursor for O–H bond activation. Subsequently, cleavage of the O–H bond of HCOOH occurs *via* transition state ^5^TS1, where the O–H bond is elongated to 1.14 Å while the C–H bond is simultaneously formed with a bond length of 1.56 Å. Meanwhile, the Cr–O bond distance shortens to 2.09 Å, indicating enhanced interaction between the formate moiety and the Cr center. These structural changes suggests that O–H bond cleavage and the formation of the C–H and Cr–O bonds proceed in a concerted manner.

Passing through ^5^TS1 leads to the formation of a stable formate intermediate ^5^IM3, in which the hydrogen atom from the hydroxyl group is transferred to a carbon atom of the fullerene framework, while the remaining formate fragment binds to the Cr center. From ^5^IM3, cleavage of the formyl C–H bond occurs *via*^5^TS2, during which the second hydrogen atom migrates to the Cr center, yielding ^5^IM4, a CO_2_-coordinated Cr–H hydride species. Subsequently, direct H_2_ formation proceeds through ^5^TS3, accompanied by regeneration of the initial catalyst ^5^C_19_Cr.

Our calculations shows that ^5^TS3 possesses the highest relative energy (38.2 kcal mol^−1^) along this pathway, identifying it as the rate-determining transition state. Consequently, an overall energy barrier of 38.2 kcal mol^−1^ is determined for the dehydrogenation of HCOOH *via* the O–H bond-first activation mechanism, rendering this pathway kinetically unfavorable under ambient conditions.

Although CO_2_ does not directly participate in H–H bond formation in ^5^TS3, removal of CO_2_ coordination results in a significantly higher activation barrier (41.9 kcal mol^−1^, Fig. S2), indicating that CO_2_ is not a mere spectator.

**Fig. 4:**
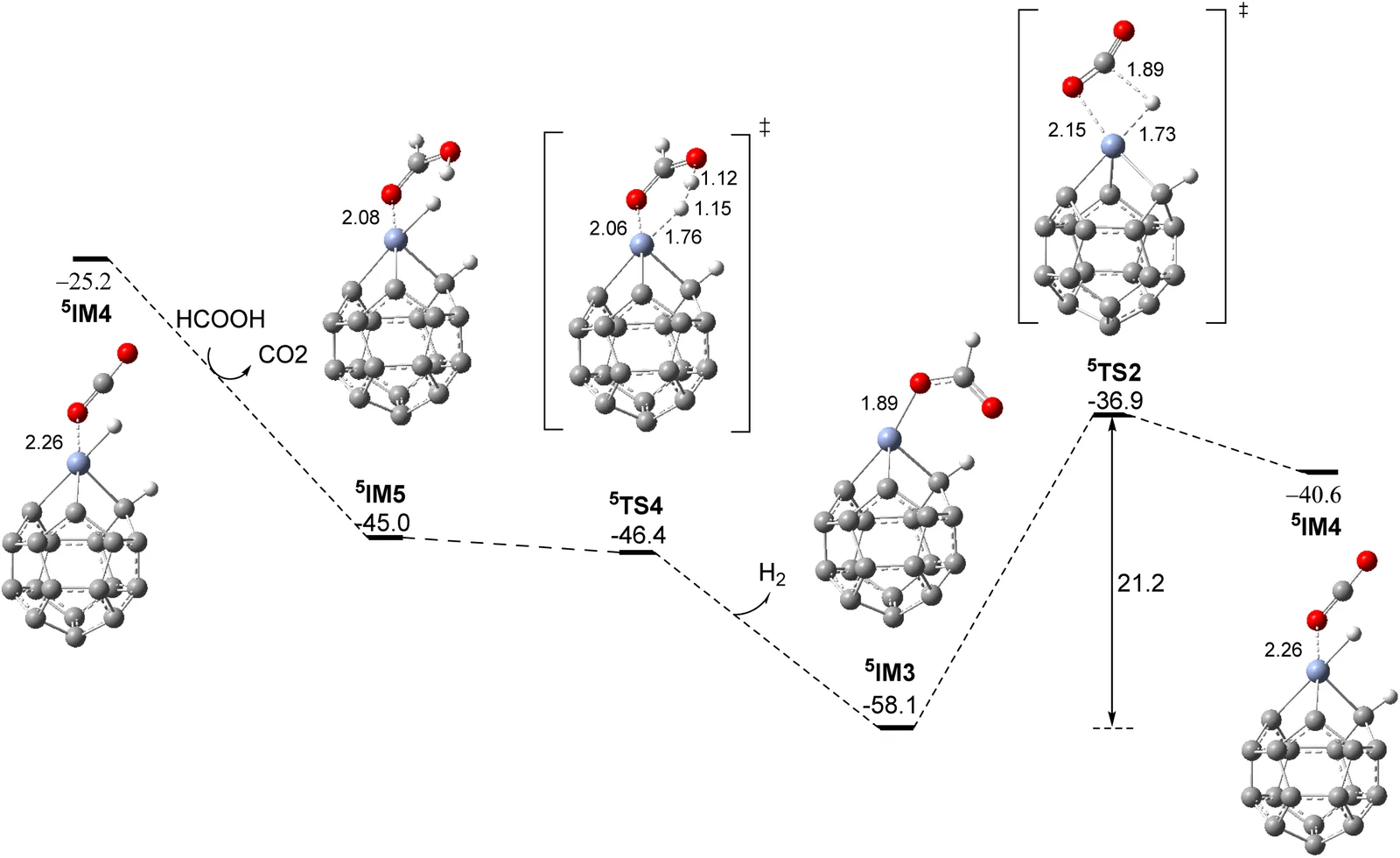
Calculated Gibbs energy profile and optimized structures for the H_2_ release assisted by a second HCOOH molecule. Relative energies are given in kcal mol^−1^, and selected bond lengths are in Å.

Given that the high activation barrier associated with direct H_2_ formation from the Cr–H hydride species ^5^IM4, alternative reaction pathways were further explored. Inspired by the work of Sirijaraensre,^43^ an energetically feasible pathway for H_2_ formation starting from ^5^IM4 was identified, and the calculated results are presented in Fig. 4. Instead of direct H_2_ formation, an additional HCOOH molecule substitutes the coordinated CO_2_ and binds to the Cr center to form ^5^IM5. Subsequent H_2_ formation proceeds *via* an essentially barrier-free transition state ^5^TS4, in which the hydroxyl hydrogen from the second HCOOH combines with the hydride hydrogen on the Cr center to form a H_2_ molecule. The resulting formate intermediate ^5^IM3 then undergoes C–H cleavage *via*^5^TS2 to regenerate ^5^IM4, thereby initiating the next catalytic cycle.

Fig. 4 clearly shows that the CO_2_-coordinated Cr–H hydride species ^5^IM4 serves as the catalytically active species in this reaction. Notably, the formation of Cr–H hydride species (^5^IM4) is identified as the rate-determining step of this pathway, with an overall energy barrier of 21.2 kcal mol^−1^. This barrier is substantially lower than that of the direct H_2_ formation pathway discussed above (Fig. 3), indicating that the HCOOH-assisted pathway is kinetically more favorable under mild reaction conditions.

#### 3.2.2 C–H bond-first activation mechanism

**Fig. 5:**
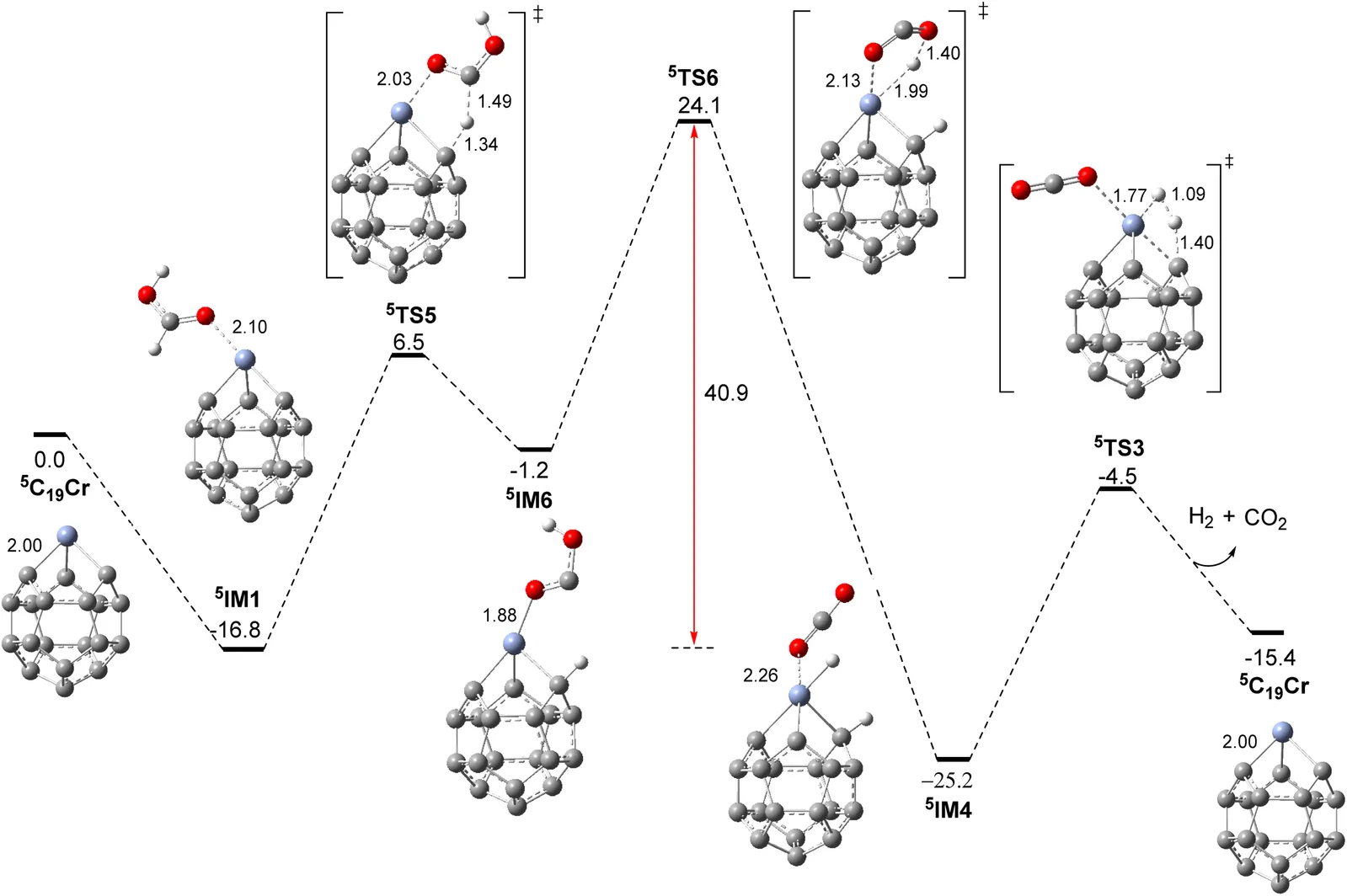
Calculated Gibbs energy profile and optimized structures for the dehydrogenation of HCOOH on ^5^C_19_Cr *via* C–H bond-first activation mechanism. Relative energies are given in kcal mol^−1^, and selected bond lengths are in Å.

Fig. 5 illustrates the reaction pathway involving initial C–H bond activation followed by O–H bond cleavage leading to H_2_ formation. Starting from ^5^IM1, the C–H bond of HCOOH is activated *via* transition state ^5^TS5, resulting in the formation of ^5^IM6. Subsequently, ^5^IM6 undergoes O–H bond cleavage *via*^5^TS6, yielding the CO_2_-coordinated Cr–H hydride species ^5^IM4. H_2_ formation then proceeds *via* transition state ^5^TS3, accompanied by the release of H_2_ and CO_2_ and regeneration of the initial catalyst, ^5^C_19_Cr.

In this pathway, formation of the catalytically active Cr–H hydride species ^5^IM4 is the rate-determining step with an energy barrier of 40.9 kcal mol^−1^, which is substantially higher than that associated with the O–H bond-first activation pathway discussed above. Consequently, the C–H bond-first activation pathway is kinetically unfavorable and can be excluded from further consideration.

### 3.3 Dehydration of HCOOH to H_2_O and CO

**Fig. 6:**
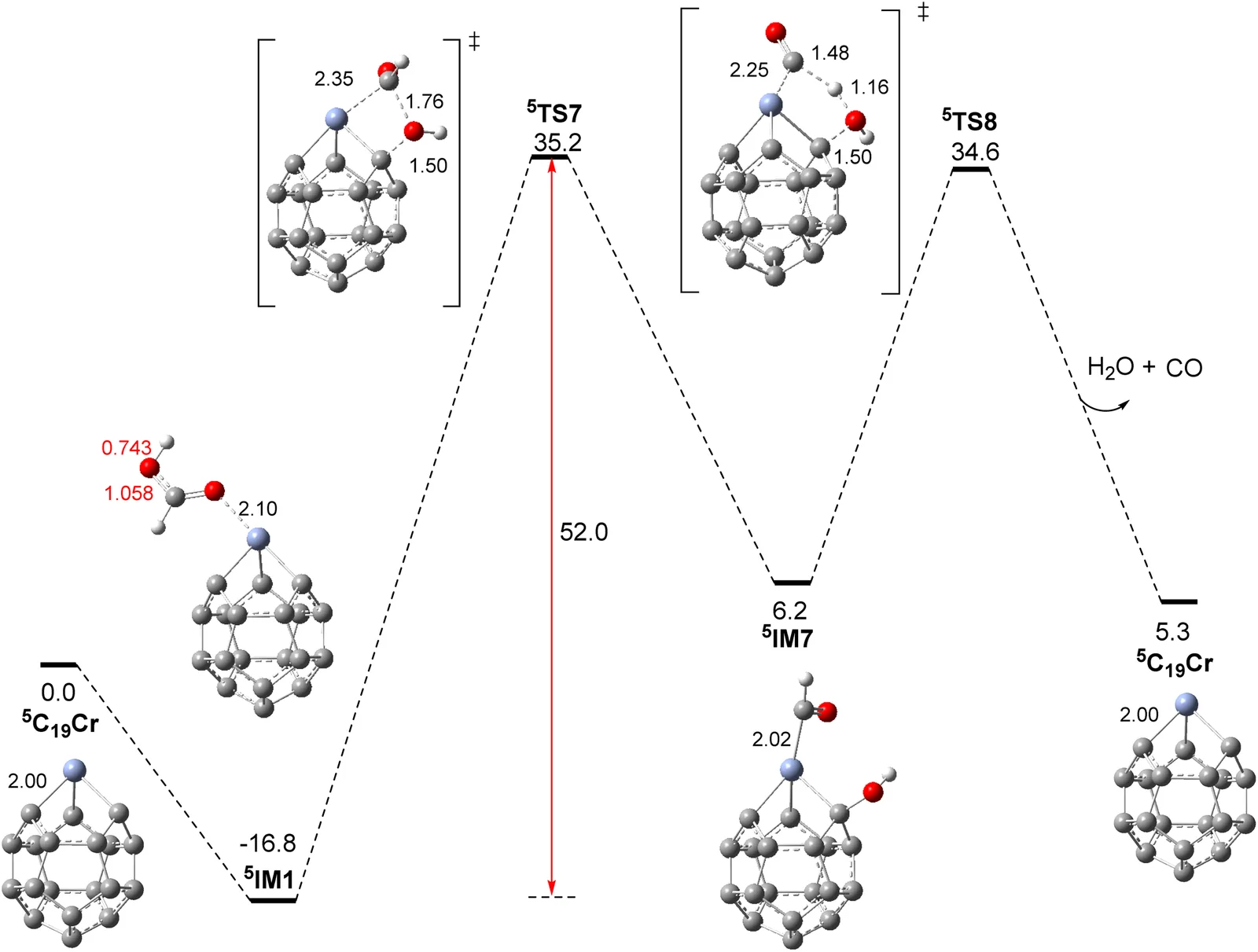
Calculated Gibbs energy profile and optimized structures for the HCOOH dehydration initiated by C–OH bond-first activation. Relative energies are given in kcal mol^−1^, selected bond lengths are in Å. Wiberg bond orders are depicted with red color.

**Fig. 7:**
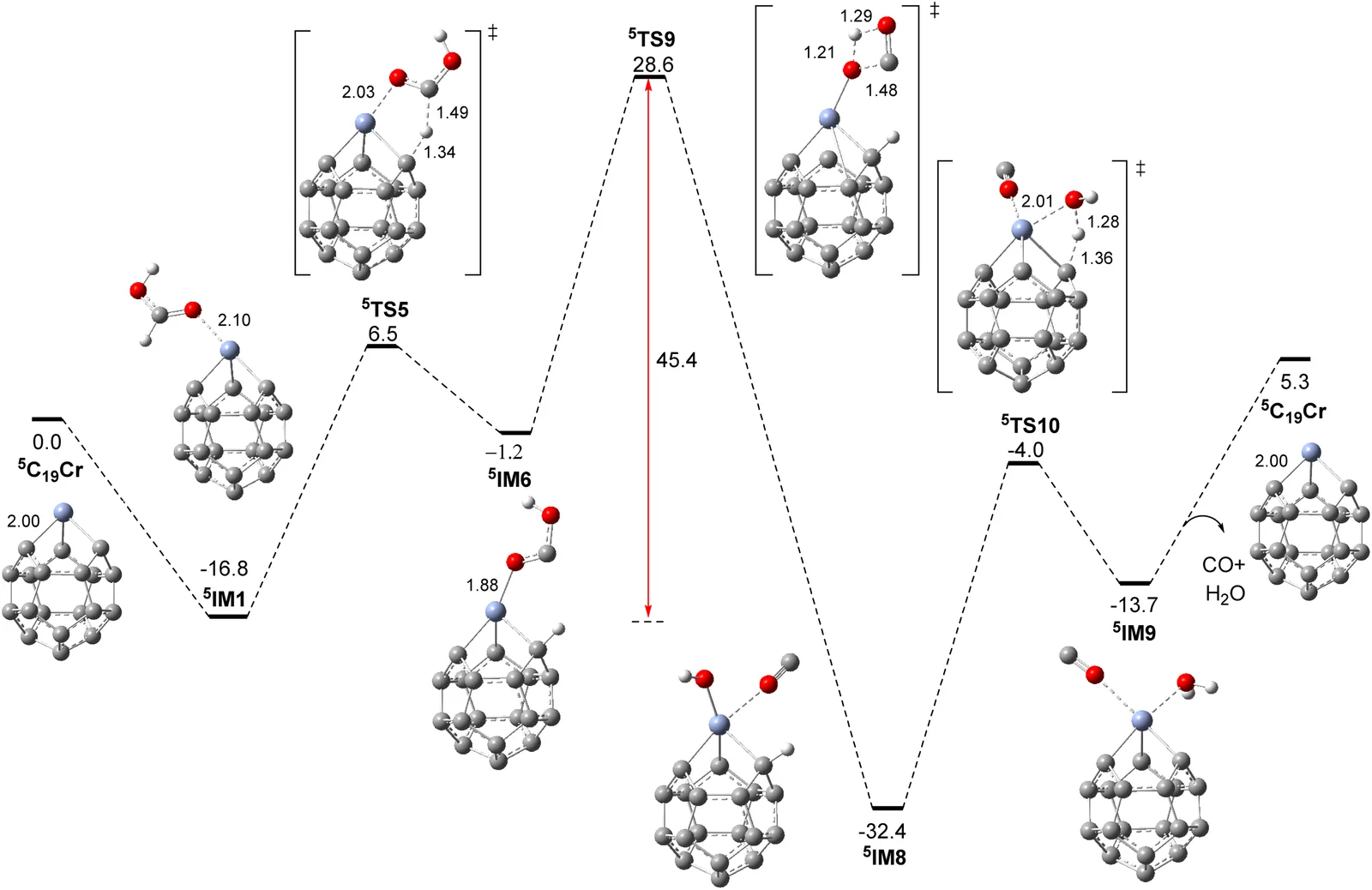
Calculated Gibbs energy profile and optimized structures for the HCOOH dehydration initiated by C–H bond-first activation. Relative energies are given in kcal mol^−1^, selected bond lengths are in Å.

In addition to the desired dehydrogenation pathway yielding H_2_ and CO_2_, HCOOH can also undergo dehydration to produce H_2_O and CO. To evaluate the feasibility of this competing reaction, the dehydration mechanism of HCOOH on ^5^C_19_Cr was investigated, and the calculated results are shown in Fig. 6 and 7.

From a mechanistic perspective, the dehydration of HCOOH to form H_2_O and CO inherently involves cleavage of both the C–OH bond and the C–H bond. Similar to the dehydrogenation reaction, dehydration can therefore proceed through different activation sequences, depending on which bond is cleaved first. Specifically, two possible dehydration pathways can be envisaged: one pathway initiated by C–OH bond cleavage, and the other initiated by C–H bond cleavage.

To assess the feasibility of these competing dehydration routes on ^5^C_19_Cr, both pathways were examined in detail, and the corresponding reaction mechanisms and energy profiles are discussed below.

#### 3.3.1 Dehydration pathway initiated by C–OH bond-first activation

Starting from the adsorbed intermediate ^5^IM1, dehydration proceeds *via* activation of the C–OH bond through transition state ^5^TS7, in which the hydroxyl group of HCOOH is transferred to a carbon atom of the fullerene framework. This elementary step is identified as the rate-determining step, which requires overcoming a barrier as high as 52.0 kcal mol^−1^, indicating that C–OH bond cleavage is kinetically unfavorable on ^5^C_19_Cr. The lower propensity for C–OH bond cleavage is further supported by Wiberg bond order analysis. In ^5^IM1, the C–OH bond exhibits a Wiberg bond order of 1.058, which is significantly higher than that of the O–H bond (0.743), indicating that the C–OH bond is substantially stronger and therefore much less susceptible to cleavage.

Following ^5^TS7, the resulting intermediate ^5^IM7 undergoes hydrogen atom transfer from the formyl group (–CHO) to the hydroxyl group *via*^5^TS8, leading to the formation of H_2_O and CO and regeneration of the ^5^C_19_Cr catalyst.

Due to the prohibitively high activation barrier associated with the C–OH bond cleavage step, the dehydration pathway of HCOOH to H_2_O and CO is kinetically inaccessible under typical reaction conditions. This result indicates the high selectivity of ^5^C_19_Cr toward HCOOH dehydrogenation.

#### 3.3.2 Dehydration pathway initiated by C–H bond-first activation

The calculated results along this pathway are shown in Fig. 7. As discussed above in Fig. 5, the formyl C–H bond of HCOOH is cleaved *via* transition state ^5^TS5, requiring an activation barrier of 23.3 kcal mol^−1^ and leading to the corresponding intermediate ^5^IM6. The following step is CO formation *via* transition state ^5^TS9, in which the hydrogen atom of hydroxyl group transfers to the carbonyl oxygen atom. This step requires an energy barrier of 45.4 kcal mol^−1^, determining the rate of this pathway. The generating CO-coordinated Cr–OH species ^5^IM8 then proceeds *via*^5^TS10, leading to the formation of ^5^IM9. Finally, the catalytic cycle is closed form ^5^IM9 with the release of CO and H_2_O and regeneration of the initial catalyst, ^5^C_19_Cr. The overall energy barrier of 45.4 kcal mol^−1^ involved indicates that the dehydration of HCOOH to H_2_O and CO *via* C–H bond activation pathway is inaccessible.

### 3.4 Overall mechanistic insights

**Fig. 8:**
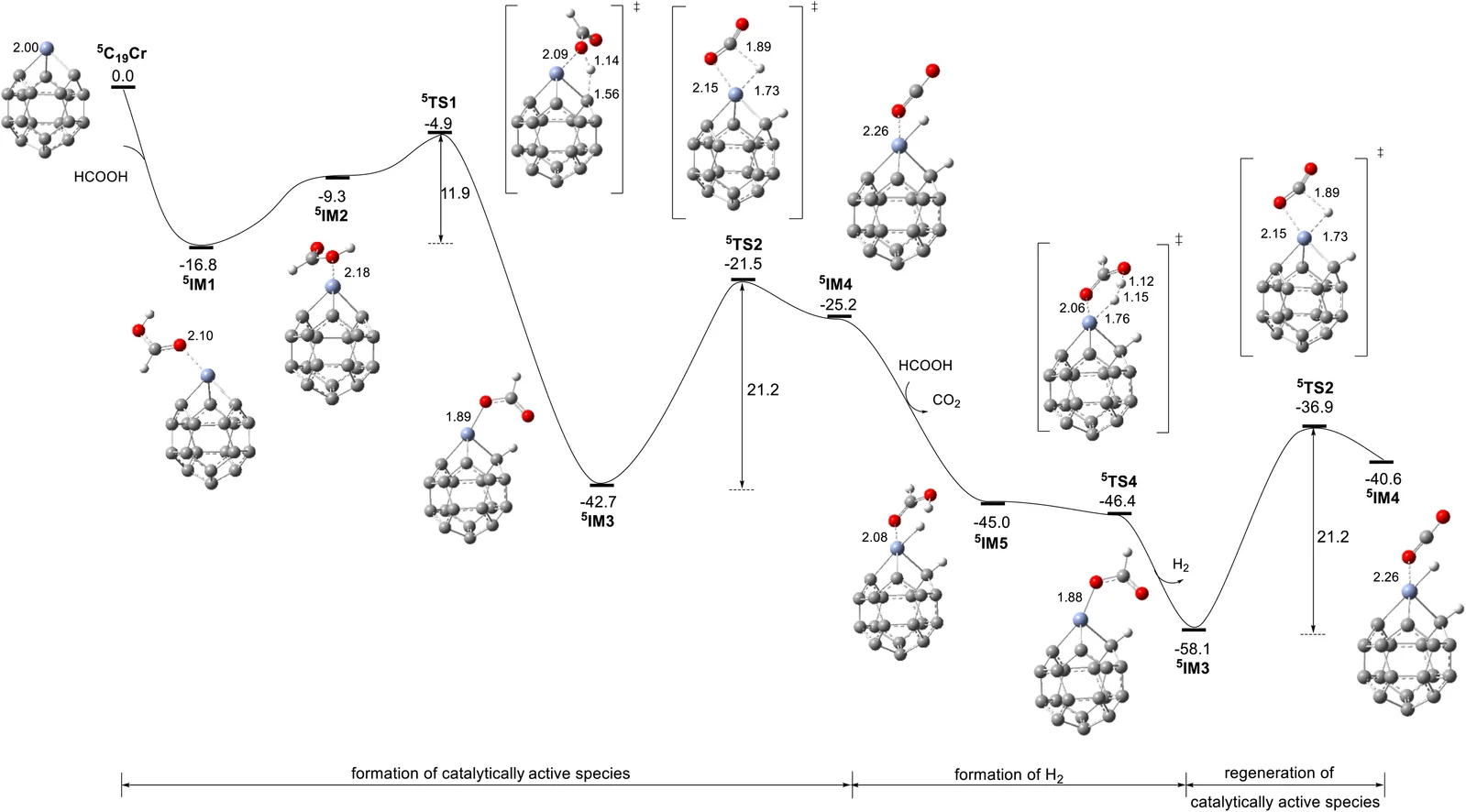
Complete Gibbs energy profile for the dehydrogenation of HCOOH to H_2_ over ^5^C_19_Cr according to the accessible mechanism.

Based on the above results, the overall mechanism of HCOOH dehydrogenation and selectivity of HCOOH decomposition over ^5^C_19_Cr are summarized in Fig. 8. The reaction is initiated by absorption of HCOOH to ^5^C_19_Cr to form ^5^IM1, which immediately converts to ^5^IM2, followed by preferential activation of the O–H bond to generate a formate intermediate ^5^IM3. Subsequent C–H bond cleavage leads to the formation of a CO_2_-coordinated Cr–H hydride species ^5^IM4. Although direct H–H coupling from ^5^IM4 is kinetically unfavorable, the formation of ^5^IM4 represents a critical step, as it serves as the true catalytically active species in the reaction. A feasible catalytic cycle is established upon participation of a second HCOOH molecule. In this pathway, the coordinated CO_2_ in ^5^IM4 is substituted by an additional HCOOH molecule to form ^5^IM5 followed by H–H coupling between the O–H hydrogen of the second HCOOH and the Cr–H hydride, leading to H_2_ release and regeneration of the formate intermediate. Subsequent C–H bond cleavage regenerates ^5^IM4, thereby completing a continuous catalytic cycle for H_2_ production.

Importantly, the reaction strongly favors dehydrogenation over dehydration on ^5^C_19_Cr. Both C–OH bond-first and C–H bond-first dehydration pathways involve prohibitively high activation barriers, rendering the formation of H_2_O and CO kinetically inaccessible. In contrast, the dehydrogenation pathway proceeds through substantially lower energy barriers enabled by the formation and utilization of the Cr–H hydride intermediate. Consequently, the preferential O–H bond activation combined with the suppression of C–OH bond cleavage accounts for the high selectivity of ^5^C_19_Cr toward HCOOH dehydrogenation.

We also explored the HCOOH dehydrogenation and dehydration pathways over triplet C_19_Cr, with all corresponding energy profiles summarized in Fig. S3–S7. The overall reaction trends and elementary energy barriers calculated on the triplet potential energy surface are similarly to those of quintet C_19_Cr. For both spin states, dehydrogenation remains kinetically more favorable than dehydration, and the relative heights of key transition-state barriers show little divergence. Given the nearly identical reaction characteristics and comparable energy barriers, quintet C_19_Cr is adopted as the primary research object for detailed mechanistic discussion in the main text.

In additional, it is worth noting that there are quantitative discrepancies between our calculated overall reaction free energies and the experimental thermodynamic values listed in Scheme 1 for both formic acid dehydrogenation and dehydration. Nevertheless, the core conclusions of this work are drawn from kinetic indicators, namely the calculated reaction energy barriers, rather than the overall thermodynamic energies of the reactions. These minor numerical differences do not undermine the reliability of our main findings.

Finally, to elucidate the origin of the enhanced catalytic performance of C_19_Cr, the adsorption behaviors of HCOOH on C_19_Cr and pristine C_20_ were systematically compared. As illustrated in Fig. S8, HCOOH adsorbs much more strongly on C_19_Cr, with an adsorption energy of −16.8 kcal mol^−1^, whereas adsorption on pristine C_20_ is thermodynamically unfavorable, giving a positive adsorption energy of 5.5 kcal mol^−1^. NBO charge analysis further reveals substantially greater charge transfer in the C_19_Cr–HCOOH complex than in the C_20_–HCOOH complex, indicating stronger electronic interactions between HCOOH and the Cr active site. These results suggest that Cr incorporation effectively modifies the electronic structure of the fullerene, thereby strengthening the adsorption and activation of HCOOH. This enhanced electronic interaction provides the fundamental origin of the superior catalytic performance of the C_19_Cr catalyst.

## 4 Conclusion

DFT calculations were performed to examine the feasibility of Cr-doped fullerenes as catalysts for formic acid dehydrogenation. The results confirm that substitutional Cr doping in a C_20_ fullerene framework enables efficient and selective HCOOH dehydrogenation, validating the initial hypothesis regarding the catalytic potential of this system. Mechanistically, HCOOH dehydrogenation proceeds preferentially *via* an O–H bond-first activation pathway, followed by C–H bond cleavage, leading to the formation of a Cr–H hydride intermediate. This hydride species acts as the true catalytically active center and enables H_2_ formation through reaction with a second HCOOH molecule. The moderate activation barrier of 21.2 kcal mol^−1^ indicates that Cr-doped fullerenes are kinetically viable catalysts under mild conditions. In contrast, dehydration pathways are associated with prohibitively high activation barriers, rendering the formation of H_2_O and CO kinetically inaccessible. As a result, HCOOH decomposition on Cr-doped fullerenes exhibits high selectivity toward dehydrogenation. Overall, this study establishes Cr-doped fullerenes as promising non-noble-metal catalysts for selective formic acid dehydrogenation and provides clear mechanistic insights that can guide the experimental design of efficient hydrogen production catalysts.

## Author contributions

Jihong Xu performed the calculations and wrote the original draft; Aili Feng analyzed the results, Shiling Yuan and Hongjun Zhou supervised this work, and Dongju Zhang wrote and reviewed this paper.

## Conflicts of interest

The authors declare no competing interest.

## Supplementary Material

RA-016-D6RA05021B-s001

## Data Availability

All data generated and analyzed during this theoretical study are included within the article and its supplementary information files. The cartesian coordinates of all optimized structures, energy data, and related computational details that support the findings of this work are available in the supplementary information (SI) of this article. No new data were deposited in public repositories for this research. Supplementary information is available. See DOI: https://doi.org/10.1039/d6ra05021b.
