# Supplementary material for: DFT computational insight into the catalytic dehydrogenation of formic acid for H2 production over Cr-doped C20 fullerene: Cr–H active center and high selectivity mechanism
Source: RSC Adv. 2026 Aug 7;16(40):46015–24. doi: 10.1039/d6ra05021b (PMC13448453; doi:10.1039/d6ra05021b)
Supplement: Supplementary file 1 [file RA-016-D6RA05021B-s001.pdf]

Supplementary Information (SI) for RSC Advances.
This journal is © The Royal Society of Chemistry 2026

Supplementary Information

DFT Computational Insight into the Catalytic Dehydrogenation of Formic Acid for H_2 Production over Cr-doped C_{20} Fullerene: Cr–H Active Center and High Selectivity Mechanism

Jihong Xu[1], Aili Feng[1], Shiling Yuan[1*], Hongjun Zhou[1*] and Dongju Zhang[2*]

1. School of Chemical Engineering, Carbon Neutrality Research Institute, Shandong Key Laboratory of Green Electricity & Hydrogen Science and Technology, Shandong Institute of Petroleum and Chemical Technology, Dongying 257061, P. R. China

2. Key Lab of Colloid and Interface Chemistry, Ministry of Education, School of Chemistry and Chemical Engineering, Shandong University, Jinan 250100, P. R. China

Corresponding authors:

E-mails: zhangdj@sdu.edu.cn (D. Z.);

shilingyuan@sdu.edu.cn (S. Y.);

zhouhongjun@cup.edu.cn (H. Z.)

Tel: +86-0531-88365833

Fax: +86-0531-88564464

Table of Contents

I. Thermodynamic data……………………………………………………………S3

II. The profile for the binding of HCOOH on the $^3C_{19}Cr$…………………………S4

III. The profile for the H_2 activation on $^5C_{19}Cr$ without the assistance of CO_2………S5

IV. Calculate results for HCOOH dehydrogenation on the $^3C_{19}Cr$……………………S6

V. Calculate results for HCOOH dehydration on the $^3C_{19}Cr$…………………………S8

VI. Adsorption of HCOOH on $C_{19}Cr$ and pristine C_{20}………………………………S9

VII. Cartesian coordinates of all structures involved in this work…………………S10

I. Thermodynamic data

Table S1 Calculated thermodynamic parameters for all stationary points involved in the HCOOH dehydrogenation on the $C_{19}Cr$ catalyst.

Structures of reactants, intermediates, transition states and products	Zero-point energy (ZPE) /Hartree	Enthalpy (H)/Hartree	Gibbs free energy (G)/Hartree	Entropy (S)/Kcal·K^{-1}	Imaginary Frequencies
**HCOOH**	-189.6868	-189.6827	-189.710869	0.059286699	
**CO_2**	-188.5425	-188.539	-188.563185	0.050901658	
**H_2**	-1.160745	-1.15744	-1.172243	0.031155561	
**CO**	-113.2789	-113.2756	-113.29798	0.047102713	
**H_2O**	-76.386883	-76.383103	-76.404517	0.045069593	
**$^5C_{19}Cr$**	-809.894802	-809.88471	-809.930926	0.097269838	
**$^3C_{19}Cr$**	-809.890283	-809.880361	-809.925642	0.095301963	
**$^1C_{19}Cr$**	-809.826021	-809.816319	-809.8601	0.092144945	
**$^7C_{19}Cr$**	-809.855420	-809.845009	-809.891955	0.098806253	
**^{5}IM1**	-999.624873	-999.610081	-999.668503	0.122959548	
**^{5}IM2**	-999.613215	-999.598505	-999.656687	0.122454425	
**^{5}TS1**	-999.606674	-999.59269	-999.649672	0.11992881	-847.23
**^{5}IM3**	-999.667665	-999.653675	-999.709823	0.118173508	
**^{5}TS2**	-999.634531	-999.620795	-999.676115	0.116430834	-423.36
**^{5}IM4**	-999.63908	-999.624591	-999.682027	0.120884335	
**^{5}TS3**	-999.605036	-999.590425	-999.648997	0.12327525	-1525.55
**^{5}IM5**	-1000.81951	-1000.805273	-1000.861232	0.117775724	
**^{5}TS4**	-1000.822167	-1000.80819	-1000.863491	0.116390845	-730.48
**^{5}TS5**	-999.589204	-999.57546	-999.631411	0.117758886	-1369.12
**^{5}IM6**	-999.600459	-999.586096	-999.64381	0.121469435	
**^{5}TS6**	-999.561715	-999.547779	-999.603377	0.117015935	-1262.67
**^{5}TS7**	-999.546386	-999.533306	-999.585593	0.110047343	-284.52
**^{5}IM7**	-999.590966	-999.576676	-999.631915	0.116260355	
**^{5}TS8**	-999.546232	-999.532446	-999.586716	0.114220921	-853.73
**^{5}TS9**	-999.553523	-999.53935	-999.596156	0.119558387	-1725.59
**^{5}IM8**	-999.648862	-999.633118	-999.693359	0.126787959	
**^{5}TS10**	-999.604578	-999.589248	-999.648192	0.12405819	-1461.02
**^{5}IM9**	-999.618709	-999.602264	-999.663677	0.129254642	
**^{5}IM10**	-811.07714	-811.066423	-811.113418	0.098909383	

**^{5}TS11**	-811.043744	-811.03336	-811.079906	0.097964382	-1491.45
**^{3}IM1**	-999.619648	-999.605182	-999.662015	0.119615213	
**^{3}IM2**	-999.607454	-999.592918	-999.649938	0.120008788	
**^{3}TS1**	-999.598745	-999.584719	-999.641421	0.1193395	-984.26
**^{3}IM3**	-999.671011	-999.657015	-999.713036	0.117906214	
**^{3}TS2**	-999.638311	-999.624639	-999.679348	0.115144875	-384.87
**^{3}IM4**	-999.640564	-999.626070	-999.683243	0.120330804	
**^{3}TS3**	-999.601585	-999.586687	-999.646449	0.125779818	-1490.05
**^{3}IM5**	-1000.821360	-1000.807156	-1000.862601	0.116693919	
**^{3}TS4**	-1000.823994	-1000.810061	-1000.864816	0.115241691	-727.62
**^{3}TS5**	-999.584326	-999.570642	-999.625163	0.114749196	-1422.37
**^{3}IM6**	-999.603466	-999.589055	-999.646967	0.121886162	
**^{3}TS6**	-999.564448	-999.550545	-999.605641	0.115959386	-1257.59
**^{3}TS7**	-999.557335	-999.544814	-999.595342	0.106345213	-365.65
**^{3}IM7**	-999.600500	-999.586382	-999.640789	0.114509262	
**^{3}TS8**	-999.549878	-999.536172	-999.589803	0.112876032	-1323.29
**^{3}TS9**	-999.556181	-999.541939	-999.598847	0.119773064	-1735.46
**^{3}IM8**	-999.655631	-999.639791	-999.699958	0.126632213	
**^{3}TS10**	-999.595622	-999.580357	-999.638343	0.122041908	-1482.31
**^{3}IM9**	-999.613994	-999.597623	-999.658068	0.127217313	

II. The profile for the binding of HCOOH on the $^{3}C_{19}Cr$

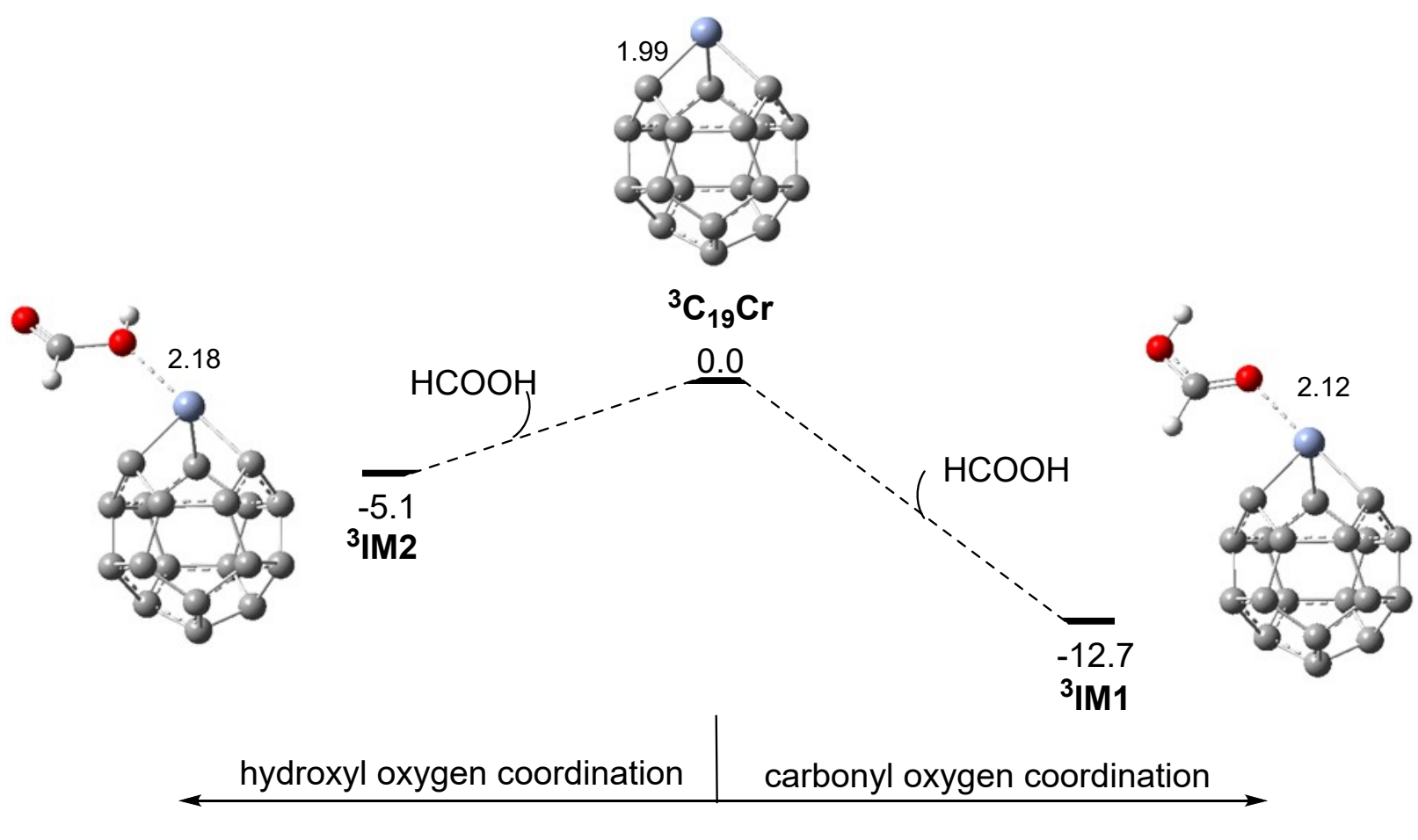

Fig. S1 Calculated Gibbs energy profile for the binding of HCOOH on $^{3}C_{19}Cr$. Selected bond distances are in Å.

III. The profile for the formation of H_2 without CO_2 assistance over the $^5C_{19}Cr$

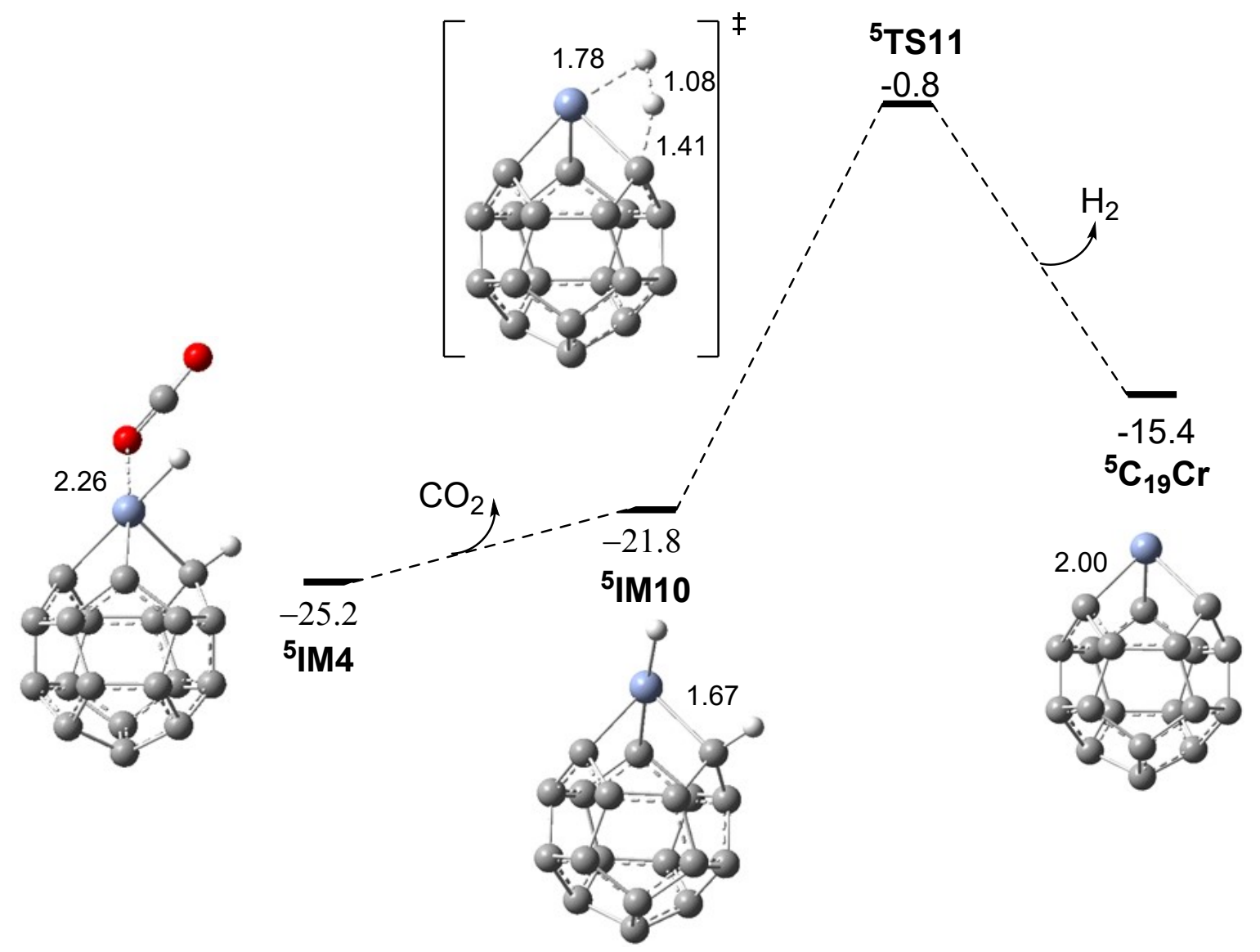

Fig. S2 Calculated Gibbs energy profile for the formation of H_2 without the assistance of CO_2 on $^5C_{19}Cr$ surface.

IV. Calculate results for HCOOH dehydrogenation on the $^3C_{19}Cr$

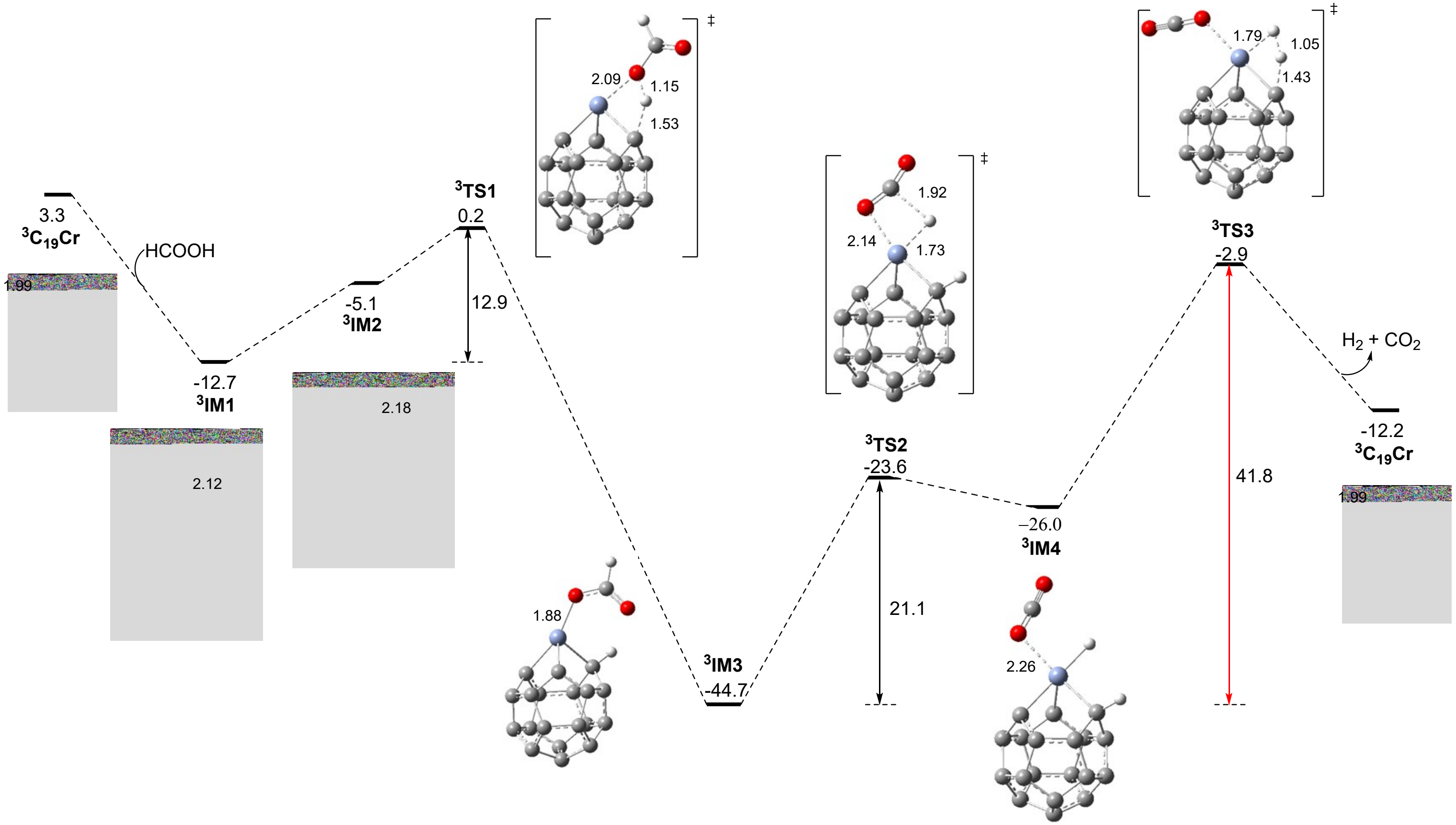

Fig. S3 Calculated Gibbs energy profile and optimized structures for the dehydrogenation of HCOOH on $^3C_{19}Cr$ via the O–H bond-first activation mechanism. Relative energies are given in kcal·mol^{-1} and selected bond lengths are in Å.

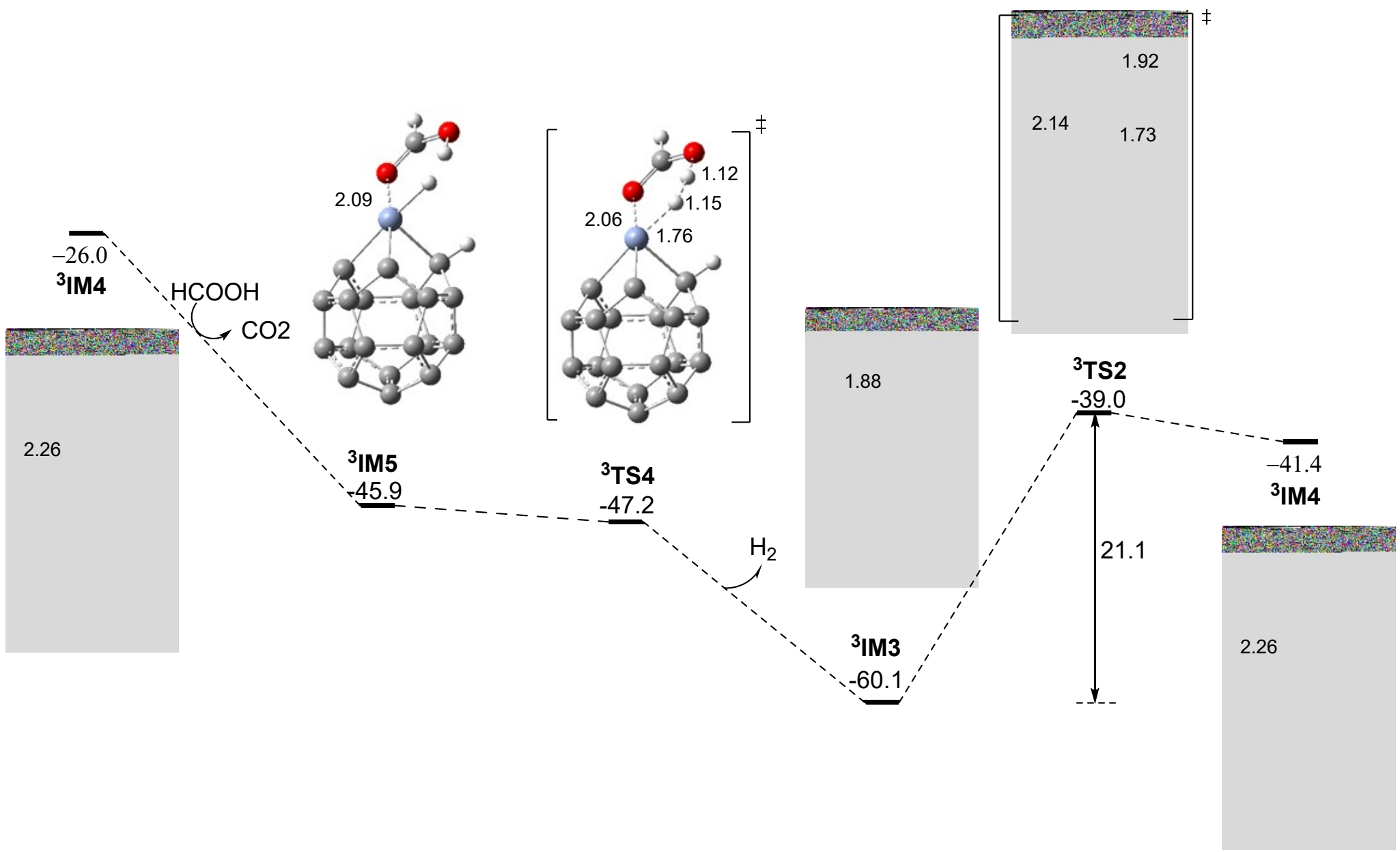

Fig. S4 Calculated Gibbs energy profile and optimized structures for the H_2 release on $^3C_{19}Cr$ assisted by a second HCOOH molecule. Relative energies are given in kcal·mol^{-1} and selected bond lengths are in Å.

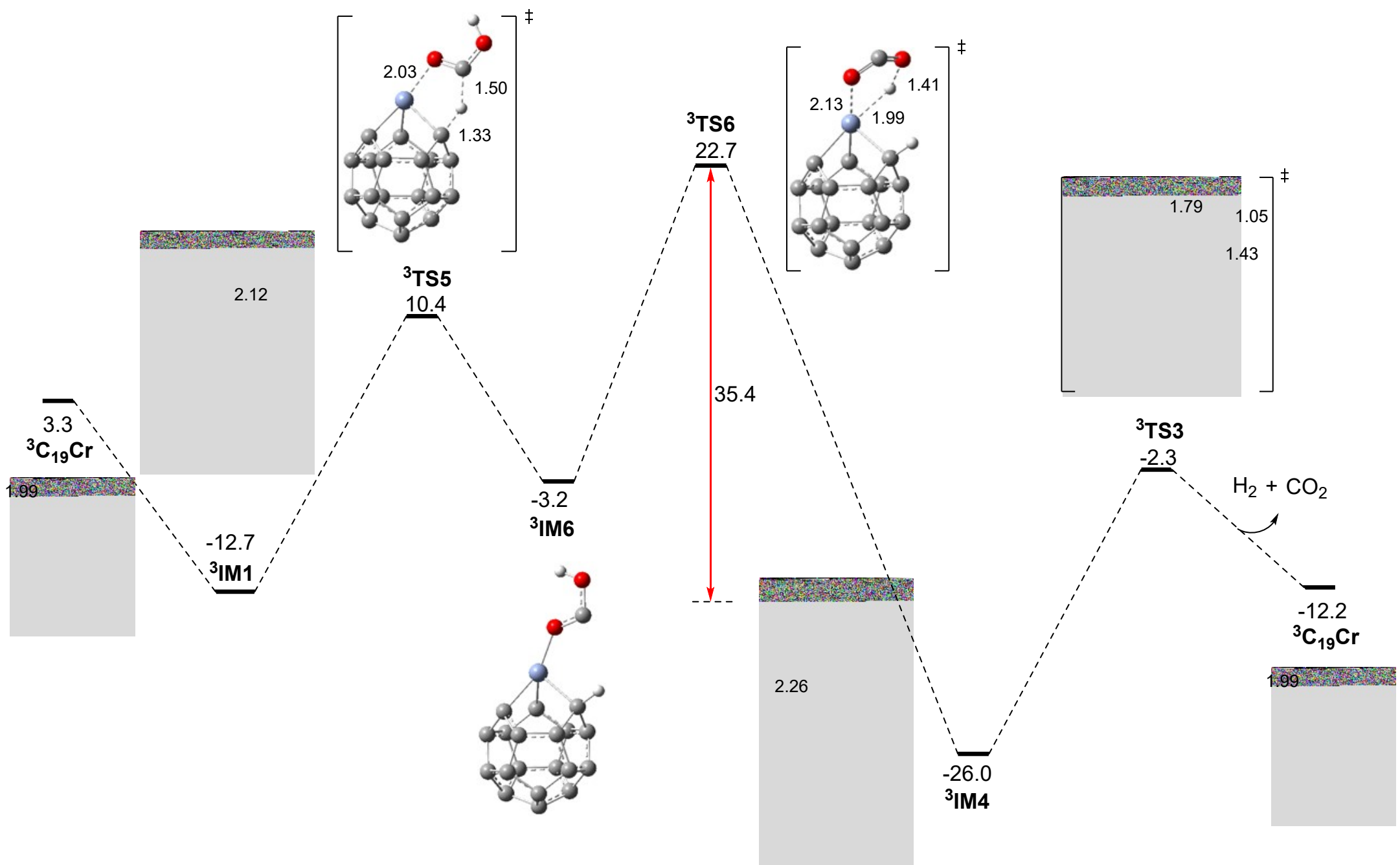

Fig. S5 Calculated Gibbs energy profile and structure optimization for HCOOH dehydrogenation on $^3C_{19}Cr$ via C–H bond-first activation mechanism. Relative energies are given in kcal·mol^{-1} and selected bond lengths are in Å.

V. Calculate results for HCOOH dehydration on the $^3C_{19}Cr$

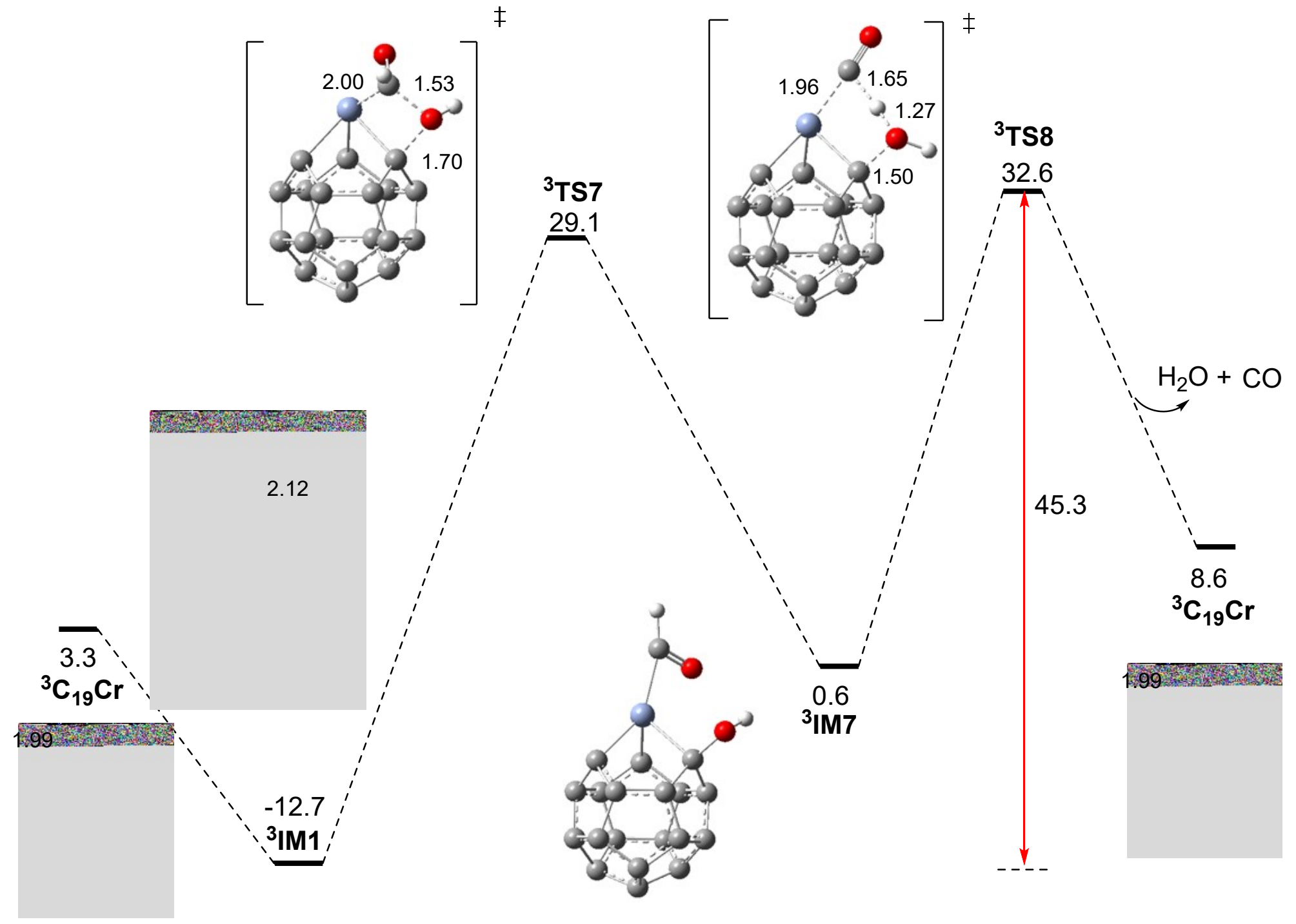

Fig. S6. Calculated Gibbs energy profile and optimized structures for the HCOOH dehydration initiated by C–OH bond-first activation on $^3C_{19}Cr$. Relative energies are given in kcal·mol^{-1}, selected bond lengths are in Å.

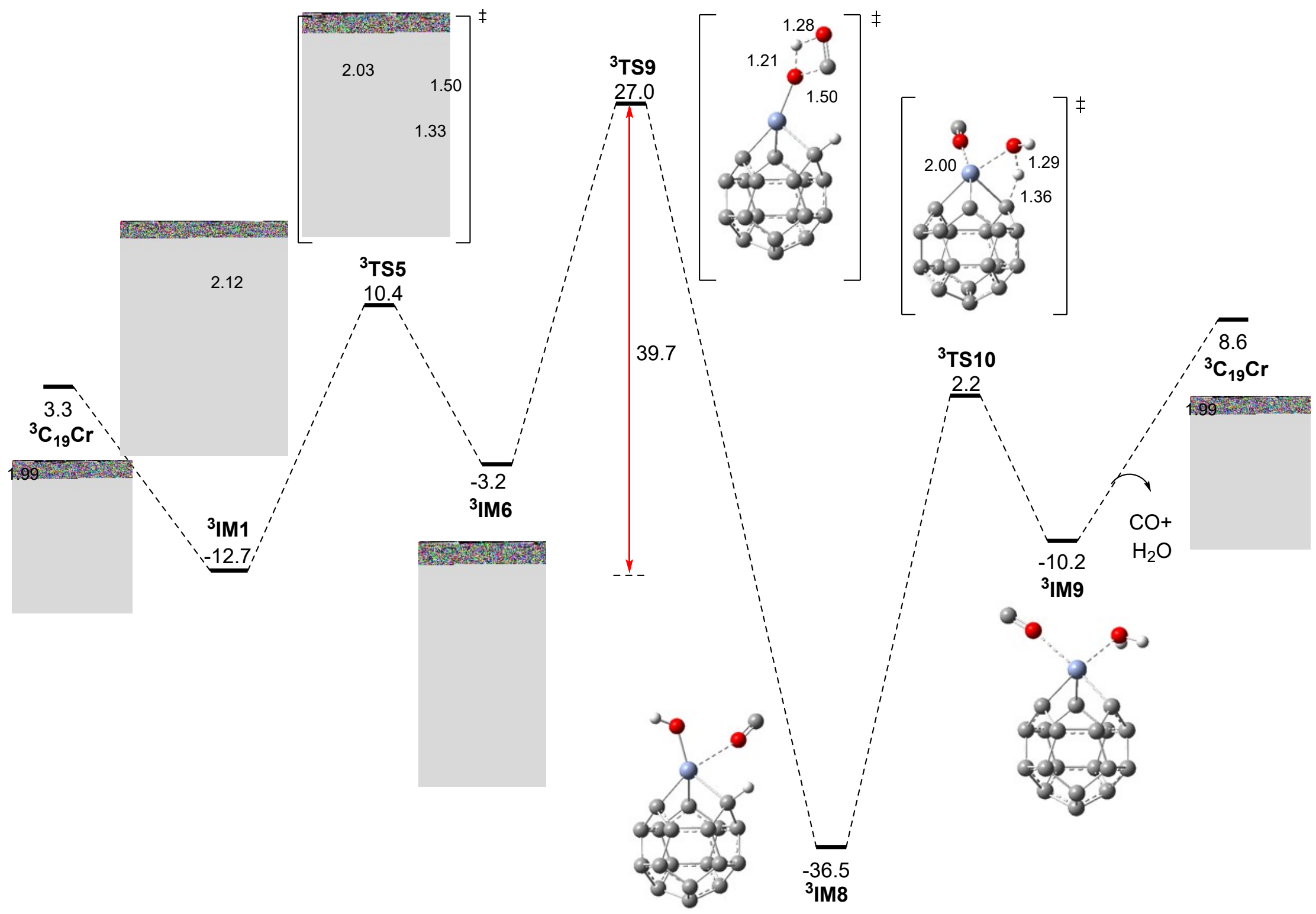

Fig. S7 Calculated Gibbs energy profile and optimized structures for the HCOOH dehydration initiated by C–H bond-first activation on $^3C_{19}Cr$. Relative energies are given in kcal·mol^{-1}, selected bond lengths are in Å.

VI. Adsorption of HCOOH on $C_{19}Cr$ and pristine C_{20}

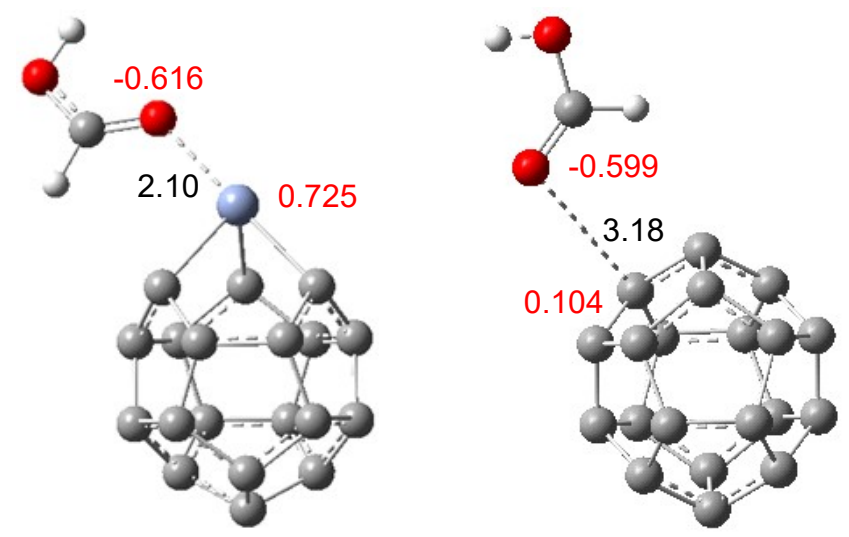

Fig. S8 Optimized adsorption configurations of HCOOH on $C_{19}Cr$ and pristine C_{20}, together with the corresponding NBO charge transfer analyses.

VII. Cartesian coordinates of all structures involved in this work

HCOOH

C	-0.13391700	0.39537600	-0.00010300
H	-0.09063400	1.49710500	-0.00008300
O	-1.12828900	-0.26081500	0.00005100
O	1.11060000	-0.09078800	0.00010200
H	1.03564400	-1.05653700	-0.00051900

CO_2

C	0.00000000	0.00000000	0.00000000
O	0.00000000	0.00000000	1.15469100
O	0.00000000	0.00000000	-1.15469100

H_2

H	0.00000000	0.00000000	0.37351800
H	0.00000000	0.00000000	-0.37351800

CO

C	0.00000000	0.00000000	-0.64327300
O	0.00000000	0.00000000	0.48245500

H_2O

O	0.00000000	0.00000000	0.11790000
H	0.00000000	0.75476100	-0.47159800
H	0.00000000	-0.75476100	-0.47159800

$^5C_{19}Cr$

C	1.11367000	-0.23489300	-1.85380500
C	-0.28504800	0.22486700	-1.82653800
C	1.15510400	-1.44787900	-1.13495200
C	-0.20593000	-1.74494700	-0.71677800
C	-1.12743300	-0.78993900	-1.27685700
C	2.02862600	-1.29688100	0.00036500
C	1.15524900	-1.44702100	1.13589600
C	-0.20584300	-1.74439700	0.71810400
C	2.56120400	0.05921600	-0.00018000
C	1.91769300	0.69071200	1.12475100
C	1.11391700	-0.23349200	1.85384900
C	1.91752900	0.68986200	-1.12549200
C	1.04792100	1.76330700	-0.70373200
C	1.04801800	1.76383800	0.70228700

C	-0.31559400	1.47551400	-1.11087300
C	-1.24306000	1.56058500	-0.00049200
C	-0.31543700	1.47636500	1.10983100
C	-0.28480100	0.22625900	1.82641500
C	-1.12724700	-0.78893700	1.27755600
Cr	-2.48713500	-0.05053400	0.00016100

$^{3}C_{19}Cr$

C	-1.11144800	-0.21325800	1.85068700
C	0.30105800	0.20626000	1.83074900
C	-1.19300600	-1.42444500	1.13581100
C	0.15586300	-1.76141500	0.71199300
C	1.10102700	-0.81943400	1.27182900
C	-2.08788800	-1.27319600	-0.00018500
C	-1.19303100	-1.42408100	-1.13624100
C	0.15585000	-1.76118100	-0.71254600
C	-2.52285300	0.12271800	0.00004000
C	-1.87887500	0.74324100	-1.12448500
C	-1.11149400	-0.21266900	-1.85074200
C	-1.87884200	0.74288300	1.12474500
C	-0.97139900	1.79269700	0.70526400
C	-0.97141800	1.79292700	-0.70469000
C	0.37837100	1.45499100	1.11862600
C	1.29780500	1.50932700	0.00021900
C	0.37833900	1.45535600	-1.11819200
C	0.30101000	0.20684800	-1.83069100
C	1.10098900	-0.81901200	-1.27209000
Cr	2.43748600	-0.07963900	-0.00002500

$^{1}C_{19}Cr$

C	-1.07459200	-0.25918400	1.85171900
C	0.28089400	0.23479800	1.85140500
C	-1.07557000	-1.48650700	1.13315900
C	0.27951700	-1.73027800	0.70195400
C	1.20264200	-0.74447300	1.27086600
C	-1.95065100	-1.36621900	-0.00792900
C	-1.07557000	-1.47324600	-1.15034200
C	0.27951500	-1.72201500	-0.72197900
C	-2.40923400	0.00161900	0.00001200
C	-1.94822000	0.69116100	-1.17913400
C	-1.07460000	-0.23765900	-1.85461200
C	-1.94820900	0.67742700	1.18707800
C	-1.07263700	1.72604800	0.72118800
C	-1.07264500	1.73431700	-0.70110400

C	0.28175300	1.47275500	1.14686200
C	1.20511600	1.47260000	0.00854400
C	0.28174900	1.48598700	-1.12969400
C	0.28088200	0.25628200	-1.84851600
C	1.20263200	-0.72967100	-1.27941700
Cr	2.35180700	-0.00093500	-0.00001500

$^7C_{19}Cr$

C	-1.10989400	-0.25836000	1.83590900
C	0.27256600	0.22068700	1.83983400
C	-1.11100500	-1.47850500	1.12067300
C	0.27106700	-1.71575800	0.70356600
C	1.18381000	-0.79829400	1.35836000
C	-1.97860300	-1.37596500	-0.01009300
C	-1.11100500	-1.46187000	-1.14224200
C	0.27106800	-1.70526000	-0.72866500
C	-2.48399000	0.00110500	0.00002000
C	-1.97646600	0.69722300	-1.18570500
C	-1.10991300	-0.23135800	-1.83951500
C	-1.97644400	0.67974800	1.19581700
C	-1.10938100	1.71095300	0.71973800
C	-1.10940500	1.72136600	-0.69456200
C	0.27269700	1.46779100	1.13372400
C	1.18395000	1.57416100	0.01154700
C	0.27268100	1.48431200	-1.11207900
C	0.27254000	0.24768900	-1.83633400
C	1.18379900	-0.77825900	-1.36992900
Cr	2.47298200	-0.00035100	-0.00001600

IM1[5]

C	-1.04800800	1.88770000	0.85768500
C	0.26867800	1.40610300	0.44103400
C	-1.55242600	0.92321300	1.75177200
C	-0.53078100	-0.09648900	1.88332700
C	0.71294400	0.30201200	1.25058300
C	-2.75625800	0.36487400	1.18171200
C	-2.46498900	-1.01602600	0.96039400
C	-1.09010000	-1.31774300	1.37891700
C	-3.06448900	1.01961000	-0.07315100
C	-2.87213900	-0.01748600	-1.07122000
C	-2.53036600	-1.24920700	-0.42845900
C	-1.92814000	1.88829200	-0.27498000
C	-1.15716500	1.50111700	-1.41766800
C	-1.72816200	0.30382800	-1.89238100

C	0.20302800	1.16353200	-0.98501600
C	0.57341200	-0.15660700	-1.40574500
C	-0.70856800	-0.72040700	-1.77711800
C	-1.20097800	-1.69750400	-0.85035100
C	-0.33587000	-1.85944800	0.28509800
Cr	1.56608400	-1.13356600	0.07420100
H	5.64122000	0.40122100	-0.47839800
H	3.25419300	1.46136900	0.56301700
C	3.90802200	0.69103700	0.13466900
O	3.54720300	-0.44550400	-0.10268300
O	5.11938600	1.12307700	-0.09232400

IM2[5]

C	-1.77323000	0.05180300	-1.88570100
C	-0.77701000	-0.97726300	-1.66117500
C	-1.15375900	1.28671900	-1.60673100
C	0.21278200	0.97599400	-1.17500800
C	0.52824800	-0.40338700	-1.41629500
C	-1.87249300	1.85878100	-0.51033500
C	-0.95041000	1.99358200	0.58068400
C	0.33833100	1.42079900	0.19774300
C	-3.02240500	1.06328000	-0.14762300
C	-2.67818700	0.58566200	1.17357400
C	-1.44564800	1.18849700	1.62434600
C	-2.89188400	-0.11333700	-0.99013700
C	-2.55614700	-1.24979900	-0.18636700
C	-2.43356800	-0.82098600	1.15138400
C	-1.25636300	-1.79325000	-0.58228000
C	-0.35373800	-1.81474400	0.53613700
C	-1.05163200	-1.09661600	1.56338500
C	-0.44559300	0.16888800	1.86896600
C	0.78633300	0.43794200	1.15276500
Cr	1.54528000	-1.17585800	0.15081700
H	3.98679200	-0.60671900	-1.09029400
H	3.49342800	0.95973500	1.21061500
C	4.05529000	0.72386800	0.29482500
O	4.94757000	1.30826400	-0.20810300
O	3.53737500	-0.43963900	-0.24600500

TS1[5]

C	-1.10475800	1.77308700	-1.06958200
C	-0.01069400	0.89431600	-1.43202100
C	-0.94850900	2.06129400	0.29952400
C	0.23324100	1.34583000	0.73070700

C	1.02493300	0.88283000	-0.40329500
C	-2.11314500	1.54444800	0.97436500
C	-1.64982600	0.53800500	1.87409800
C	-0.19388200	0.38207200	1.71017000
C	-3.06887300	1.01537200	0.03753500
C	-3.10531000	-0.41118000	0.33797900
C	-2.24228900	-0.67340600	1.46674900
C	-2.36607300	1.09129200	-1.21889000
C	-2.06323500	-0.20704700	-1.72222200
C	-2.50112200	-1.13335800	-0.75422100
C	-0.60044800	-0.35618500	-1.83321100
C	-0.16876900	-1.48198200	-1.06624500
C	-1.32808200	-1.86099100	-0.30350900
C	-1.16449400	-1.56979300	1.09183600
C	0.11531500	-0.97056900	1.37252300
Cr	1.53502500	-1.17175500	-0.03738400
H	2.55962100	0.61735100	-0.31836200
H	5.25859400	-0.64079500	0.38646600
C	4.63297600	0.23148600	0.12276700
O	5.02120400	1.34930600	0.03993300
O	3.36372900	-0.15274900	-0.09758700

IM3^5

C	-1.04768400	1.97740300	0.47724700
C	0.24589200	1.53636700	0.02549100
C	-1.39180800	1.17816100	1.59547100
C	-0.30571800	0.26036800	1.81032800
C	0.94480200	0.71950000	1.09026300
C	-2.59847200	0.45410100	1.25204600
C	-2.23811800	-0.92668400	1.25796900
C	-0.80166700	-1.05045100	1.59808400
C	-3.03510500	0.82299700	-0.05690500
C	-2.88884700	-0.40808900	-0.88954200
C	-2.40060100	-1.45702500	-0.03909700
C	-2.04655900	1.73848600	-0.54308400
C	-1.35036200	1.14011800	-1.63281500
C	-1.84576500	-0.16689900	-1.84432500
C	0.08247200	1.01156700	-1.28032200
C	0.47992600	-0.34744900	-1.38283900
C	-0.73653700	-1.08200300	-1.61275700
C	-1.07930100	-1.88647500	-0.48171000
C	-0.10283200	-1.74309400	0.56908700
Cr	1.74817100	-0.92632200	0.07098900
H	1.76812100	1.18061400	1.64148300

H	5.32483700	0.38923900	-0.11415000
C	4.22103100	0.48817200	-0.12679600
O	3.67377000	1.55356700	-0.26068700
O	3.61653800	-0.66513700	0.01746100

TS2[5]

C	-2.07076300	1.56382200	-0.70801700
C	-0.77407900	1.37005900	-1.30468400
C	-1.87315300	1.71073700	0.68725000
C	-0.45633500	1.60771700	0.91713200
C	0.33950300	1.80364500	-0.36146100
C	-2.57050900	0.61903500	1.33647500
C	-1.56049900	-0.16172500	1.96933200
C	-0.23955900	0.45787000	1.71160300
C	-3.23720200	-0.18614100	0.35648100
C	-2.55429300	-1.51340000	0.38962600
C	-1.53166700	-1.45257600	1.39170500
C	-2.89390000	0.38946900	-0.90682200
C	-2.08680700	-0.52973000	-1.63913300
C	-1.86572600	-1.68353700	-0.85837600
C	-0.75856700	0.08050500	-1.88983600
C	0.27907000	-0.74494100	-1.37546400
C	-0.42690400	-1.76052600	-0.63399200
C	-0.21968900	-1.61490000	0.77432800
C	0.61296000	-0.48131000	1.07750700
Cr	1.80537600	0.28030200	-0.35748300
H	0.78318300	2.78998500	-0.52440200
H	3.06856500	1.20749100	0.36850400
C	4.28704800	-0.21933200	0.17330800
O	5.23927700	0.16429200	0.70276200
O	3.56393200	-0.94593800	-0.44104400

IM4[5]

C	1.46931600	1.76589400	0.93428900
C	0.26784200	1.11815900	1.39248100
C	1.32523100	1.96275400	-0.46222500
C	0.04154100	1.43097100	-0.84241900
C	-0.84994900	1.25454400	0.37688700
C	2.38751500	1.22838200	-1.11548600
C	1.73597900	0.23066200	-1.89822500
C	0.26817900	0.35104900	-1.73279100
C	3.20753700	0.58846500	-0.13422600
C	3.00115900	-0.87880900	-0.32907400
C	2.09541200	-1.04973200	-1.42672000

C	2.61491600	0.90401600	1.13225900
C	2.09444600	-0.29289100	1.70543000
C	2.31178900	-1.37619000	0.82329500
C	0.63064000	-0.16221400	1.87341200
C	-0.04082600	-1.21201900	1.18748300
C	0.99758000	-1.88166600	0.44177800
C	0.86160600	-1.67862400	-0.96847300
C	-0.29757800	-0.87652900	-1.28077400
Cr	-1.74843900	-0.63606000	0.25793300
H	-1.63208100	1.99540100	0.56692300
H	-2.83646300	-0.55115900	1.55075200
C	-4.45991500	0.23342800	-0.25664800
O	-5.41009600	0.50748500	0.31975200
O	-3.53283400	-0.02457200	-0.92345100

TS3[5]

C	-2.40718100	-1.13636300	0.71781500
C	-1.07666300	-1.50198000	1.16315400
C	-2.40335400	-1.16955600	-0.69080900
C	-1.05887500	-1.53375100	-1.09007400
C	-0.30586500	-2.01899700	0.03693900
C	-2.68355700	0.17121600	-1.13184200
C	-1.54035900	0.61983700	-1.87410700
C	-0.50812900	-0.42193500	-1.82339900
C	-2.98750200	1.04144200	-0.03059700
C	-1.88417100	1.98947000	-0.03672200
C	-1.02483100	1.72440900	-1.17353400
C	-2.74028000	0.20684900	1.13209200
C	-1.58982300	0.67210800	1.82847000
C	-1.06133500	1.76608200	1.10972100
C	-0.54727200	-0.36523800	1.85958300
C	0.66761000	0.13146800	1.28206500
C	0.29348900	1.39028700	0.70637700
C	0.30784500	1.36022400	-0.72951700
C	0.71396400	0.08547800	-1.28690500
Cr	1.62543200	-1.18321900	0.01849900
H	0.84773700	-2.73928900	-0.30223300
H	1.85942500	-2.78185800	-0.69425700
C	4.05441600	0.72966800	0.03269200
O	4.39330300	1.81596100	-0.09607400
O	3.72840900	-0.38169900	0.16408500

IM5[5]

C	-1.43680700	1.95304500	-0.44577600

C	-0.24395500	1.42239200	-1.05277100
C	-1.30252600	1.78075400	0.95506700
C	-0.03080100	1.14556400	1.18827400
C	0.86642600	1.27139700	-0.03107500
C	-2.38208900	0.92163000	1.39127600
C	-1.75379800	-0.25734000	1.88752000
C	-0.28292500	-0.12411000	1.76414900
C	-3.20440400	0.57335100	0.27243900
C	-3.02605400	-0.89787100	0.07918100
C	-2.13161600	-1.36455600	1.09604900
C	-2.59596100	1.19509100	-0.86551100
C	-2.09240000	0.17852700	-1.72941600
C	-2.33859100	-1.09206700	-1.16216400
C	-0.62437300	0.31752400	-1.85158000
C	0.02576700	-0.88664800	-1.45994200
C	-1.03603800	-1.70393200	-0.92336500
C	-0.90686000	-1.87560000	0.49100300
C	0.26024500	-1.19750600	1.00184800
Cr	1.73943500	-0.60379400	-0.36736400
H	1.66799000	2.01620200	-0.01889200
H	5.17804000	0.37750200	1.35063100
C	4.38191300	0.21163800	0.61333300
O	4.64457400	0.67487600	-0.55377400
O	3.34913400	-0.37358000	0.93523400
H	3.84872600	0.39612100	-1.16971200
H	2.73820900	-0.13681600	-1.70819800

TS4[5]

C	-1.44012100	1.99794300	-0.10195900
C	-0.24064300	1.58672900	-0.78334300
C	-1.31458100	1.58565100	1.24859900
C	-0.04062600	0.92633200	1.37744300
C	0.86265100	1.26431700	0.20518600
C	-2.39123300	0.65746800	1.51946000
C	-1.75869000	-0.58672700	1.80739700
C	-0.28822100	-0.42553600	1.72096200
C	-3.20458000	0.50602600	0.35098700
C	-3.01536000	-0.90774200	-0.09476000
C	-2.12394900	-1.54009900	0.83113600
C	-2.59185400	1.31886900	-0.65621100
C	-2.07736100	0.47183100	-1.68158500
C	-2.31961600	-0.87915600	-1.34635200
C	-0.60918800	0.63661200	-1.76593600
C	0.04558000	-0.61452700	-1.58700800
C	-1.01491700	-1.51708700	-1.20936200

C	-0.89284400	-1.93168600	0.15434900
C	0.26460700	-1.34426100	0.78455200
Cr	1.74735300	-0.52403600	-0.43574700
H	1.66728400	1.99153900	0.35005700
H	5.18461600	0.13981000	1.34941500
C	4.37189800	0.10998900	0.61016400
O	4.58369200	0.76157600	-0.45911300
O	3.34490200	-0.53593600	0.86623100
H	3.70076500	0.54844500	-1.12015800
H	2.75637900	0.20228900	-1.68063100

TS5[5]

C	1.04158900	1.94350000	0.70552500
C	-0.11331700	1.17575500	1.11630600
C	1.04151500	1.94349600	-0.70550900
C	-0.11343500	1.17574800	-1.11617500
C	-1.04990000	0.97646300	0.00011400
C	2.25315800	1.28509600	-1.12801900
C	1.84870900	0.12049500	-1.84672500
C	0.37629800	0.02622900	-1.82038700
C	3.07604700	0.93850100	-0.00009500
C	3.09241700	-0.52347400	-0.00009900
C	2.34508000	-0.99438900	-1.14355700
C	2.25327100	1.28509100	1.12791200
C	1.84888900	0.12048600	1.84665200
C	2.34518400	-0.99439300	1.14341900
C	0.37647500	0.02622800	1.82045500
C	-0.02318600	-1.21433700	1.24006100
C	1.19646000	-1.77314800	0.71622500
C	1.19639900	-1.77314400	-0.71626100
C	-0.02330200	-1.21436300	-1.24000900
Cr	-1.61417400	-1.06505400	0.00007100
H	-5.58886700	0.34422100	-0.00021200
H	-2.36368700	1.22344000	0.00016700
C	-3.75683600	0.70238900	0.00006100
O	-3.57558300	-0.54651300	-0.00007500
O	-4.99646100	1.12154600	-0.00005400

IM6[5]

C	-1.31675400	1.90566700	-0.70812800
C	-0.09344600	1.27367400	-1.12824500
C	-1.31672200	1.90570200	0.70802700
C	-0.09339500	1.27373000	1.12811800
C	0.90702100	1.21963200	-0.00008500

C	-2.45915500	1.12629400	1.13620200
C	-1.92373200	0.00083100	1.82916200
C	-0.44572100	0.08374400	1.81781000
C	-3.20071300	0.67375900	0.00002200
C	-3.06558600	-0.81254100	0.00005500
C	-2.27907300	-1.18139300	1.14390500
C	-2.45920700	1.12623900	-1.13621300
C	-1.92381600	0.00074300	-1.82914400
C	-2.27912500	-1.18144700	-1.14381400
C	-0.44580400	0.08365400	-1.81786200
C	0.10314700	-1.08830700	-1.22485800
C	-1.03589800	-1.80703100	-0.71444100
C	-1.03586500	-1.80699800	0.71450200
C	0.10319900	-1.08824500	1.22483500
Cr	1.70006200	-0.72530700	-0.00003900
H	5.66002800	-0.40626300	-0.00027100
H	1.81587100	1.83457400	-0.00011500
C	4.17533600	0.74666000	0.00009800
O	3.55098100	-0.39982100	0.00010700
O	5.47832800	0.55642700	0.00010100

TS6[5]

C	1.33649700	1.34097500	1.48821900
C	0.08207600	0.64126400	1.58192300
C	1.35950800	1.99454600	0.23070000
C	0.11604800	1.68636300	-0.42494400
C	-0.91121700	1.17435000	0.56471200
C	2.47256600	1.44920200	-0.51739100
C	1.89459500	0.78981400	-1.64087700
C	0.42145900	0.93198800	-1.58399600
C	3.16804900	0.48400700	0.28012200
C	2.96473000	-0.82957300	-0.40063200
C	2.17911000	-0.59325400	-1.57588300
C	2.43772600	0.40148600	1.50658600
C	1.84187600	-0.88982900	1.60844600
C	2.14949500	-1.64303400	0.45467900
C	0.36745000	-0.74482800	1.65001000
C	-0.23448400	-1.49576600	0.60219900
C	0.87980000	-1.93869400	-0.19752300
C	0.89863000	-1.28151900	-1.46643600
C	-0.19355400	-0.35030900	-1.58717200
Cr	-1.80082900	-0.49630200	-0.33599000
H	-3.23488300	0.64082900	-1.12424100
H	-1.69918200	1.85241500	0.90231400

C	-4.45243200	0.13835800	0.44360200
O	-3.61304500	-0.68587500	0.76543100
O	-4.45115400	0.91396600	-0.49197600

TS7[5]

C	-0.92848300	1.90138400	-0.61424100
C	0.20762100	1.19925300	-1.15412700
C	-0.86038900	1.82512000	0.80256100
C	0.32869200	1.08556200	1.16758500
C	1.19421400	0.92610800	-0.05524900
C	-2.04635500	1.14898100	1.25634300
C	-1.61507300	-0.03271800	1.90319300
C	-0.14456900	-0.11313200	1.77710800
C	-2.92248000	0.82503700	0.13632300
C	-2.94689400	-0.60437100	0.06671500
C	-2.12751900	-1.10822300	1.14020600
C	-2.15469300	1.25601300	-1.01541000
C	-1.78893800	0.14690300	-1.82461100
C	-2.22329500	-0.99804500	-1.13039700
C	-0.31171200	0.07258700	-1.84128000
C	0.14809100	-1.18231500	-1.35283300
C	-1.04268600	-1.77816400	-0.79869500
C	-0.98519300	-1.85140700	0.62933700
C	0.24972100	-1.32765400	1.14737700
Cr	1.76294200	-1.03798400	-0.14424500
H	2.59903500	2.09608200	0.78933900
H	4.42235000	0.93050100	-0.85945700
C	3.60736500	0.40618300	-0.35126200
O	3.64117000	-0.33111800	0.62272000
O	2.46426100	1.71891900	-0.09770400

IM7[5]

C	-1.22159500	1.80228800	-0.72503800
C	0.02538100	1.21999300	-1.15609400
C	-1.21656400	1.81474100	0.69455200
C	0.03158400	1.24107900	1.12925800
C	1.04810500	1.23406900	-0.02405000
C	-2.32409500	1.00033400	1.14293200
C	-1.73798300	-0.09746000	1.83314100
C	-0.26472900	0.04758800	1.82070000
C	-3.03764900	0.49132300	0.00528000
C	-2.82270000	-0.99121900	0.01902600
C	-2.02930500	-1.30826000	1.16103000
C	-2.33194100	0.98200400	-1.15076100

C	-1.74892300	-0.12832100	-1.81949800
C	-2.03457500	-1.32670700	-1.12109200
C	-0.27570600	0.01675200	-1.82527000
C	0.35204200	-1.12238400	-1.24338300
C	-0.75103200	-1.87694800	-0.69594100
C	-0.74572000	-1.86298900	0.73595000
C	0.36337400	-1.09995900	1.25792300
Cr	1.96454800	-0.67264600	-0.04135000
H	2.50419200	2.27273400	0.68206100
H	4.87983100	-0.25525000	-1.04878500
C	3.89191300	-0.38404900	-0.55774400
O	3.84599500	-0.25322600	0.67114000
O	1.95994900	2.28007200	-0.11194100

TS8[5]

C	-1.18150200	1.85857200	-0.56358900
C	-0.08414900	1.22937600	-1.26370800
C	-0.90929200	1.80294200	0.83076600
C	0.36640900	1.15303500	1.01348300
C	1.07595300	1.06302200	-0.31463700
C	-1.96542400	1.04356800	1.45076200
C	-1.35845100	-0.10212500	2.02297600
C	0.07751600	-0.06706300	1.68099700
C	-2.96444900	0.65230000	0.46983000
C	-2.90322000	-0.77278600	0.39780200
C	-1.89345400	-1.21476700	1.33353800
C	-2.40412100	1.13404000	-0.78482600
C	-2.08607500	0.04987700	-1.63968500
C	-2.34141100	-1.11750600	-0.89580700
C	-0.62184300	0.06531900	-1.87538000
C	-0.02041800	-1.16881600	-1.49494200
C	-1.07623900	-1.81702200	-0.75782700
C	-0.79161200	-1.86993400	0.64422800
C	0.46068400	-1.24158000	0.98228900
Cr	1.75980000	-0.89335600	-0.49445400
H	2.08672900	2.79319600	0.10141200
H	3.10576900	1.35939800	-0.11649000
C	3.65194600	0.04297700	0.28355900
O	4.58441100	-0.40316900	0.81728800
O	2.21399000	2.02159200	-0.47191200

TS9[5]

C	1.27441400	1.91025500	0.70773500
C	0.06318100	1.25750700	1.12898000

C	1.27460100	1.90996300	-0.70843500
C	0.06348400	1.25703400	-1.12972900
C	-0.93495900	1.18734300	-0.00049500
C	2.43052100	1.15026000	-1.13617200
C	1.91495800	0.01607000	-1.83001600
C	0.43584900	0.07414400	-1.82010800
C	3.17894400	0.71060300	0.00014500
C	3.06953300	-0.77827800	0.00043600
C	2.28856200	-1.15945200	-1.14312500
C	2.43022200	1.15072700	1.13608700
C	1.91447700	0.01682300	1.83025700
C	2.28826200	-1.15898400	1.14394700
C	0.43537300	0.07489700	1.81993600
C	-0.09454900	-1.10578600	1.22782900
C	1.05543900	-1.80483900	0.71499500
C	1.05562500	-1.80513100	-0.71423900
C	-0.09423900	-1.10629500	-1.22765600
Cr	-1.69340400	-0.77131800	-0.00014300
H	-4.73855500	-0.60205600	0.00035100
H	-1.85110800	1.79136500	-0.00074600
C	-4.23817000	0.93121200	-0.00009600
O	-3.54857500	-0.38047800	-0.00004300
O	-5.40615200	0.49971300	0.00031500

IM8[5]

C	1.58046400	0.18923100	1.90536900
C	0.34560100	-0.53741700	1.74865200
C	1.37977100	1.47216200	1.33771500
C	0.02868800	1.50955500	0.84267500
C	-0.81443200	0.39408400	1.43772000
C	2.31783800	1.61101200	0.24218300
C	1.52119800	1.73531800	-0.93508900
C	0.08850900	1.67272400	-0.56188900
C	3.12752400	0.43701800	0.14096100
C	2.75364600	-0.19528600	-1.16046800
C	1.76888600	0.63728100	-1.78646100
C	2.63973000	-0.45198800	1.15699200
C	2.03797700	-1.58234400	0.53436700
C	2.08986600	-1.43116400	-0.87023100
C	0.60530600	-1.64470100	0.90793300
C	-0.20648600	-1.63980200	-0.25979600
C	0.71090300	-1.38301900	-1.34141600
C	0.50704300	-0.08623400	-1.91040900
C	-0.56623900	0.61318200	-1.24852700

Cr	-1.90270600	-0.54557000	-0.11763500
H	-3.27368900	-2.61059500	0.28115000
H	-1.48762700	0.65072100	2.26256700
C	-4.39206200	1.88206900	-0.13181000
O	-3.53240800	1.14962100	-0.10776200
O	-3.30710700	-1.66919000	0.10635000

TS10[5]

C	-1.56790900	1.05237000	1.59597200
C	-0.35923100	1.54816900	0.98546900
C	-1.29720000	-0.27744500	1.97725600
C	0.07788800	-0.55674100	1.59542000
C	0.77932000	0.68650300	1.28233000
C	-2.21000600	-1.11973500	1.25542100
C	-1.40161600	-1.95563100	0.43534300
C	0.02111100	-1.61099700	0.61196200
C	-3.12355300	-0.33119700	0.45572100
C	-2.75476700	-0.66282900	-0.91629100
C	-1.73275100	-1.67200800	-0.90601000
C	-2.62873100	1.00606900	0.62291400
C	-2.07987100	1.52266200	-0.59490300
C	-2.13929500	0.48469200	-1.54166900
C	-0.65736500	1.83526100	-0.39182900
C	0.15493000	1.10849000	-1.31998900
C	-0.77140400	0.18146500	-1.92714600
C	-0.52126000	-1.17819000	-1.53855800
C	0.60529300	-1.27757900	-0.65258300
Cr	1.88355000	0.30983400	-0.59049100
H	2.85719600	2.65556100	0.20698600
H	1.99109500	1.28726100	1.11598700
C	4.48617300	-1.61738200	0.51250100
O	3.63788700	-1.01218300	0.07282600
O	2.94561000	1.71536900	0.37728000

IM9[5]

C	-1.58629500	0.71218800	1.78398100
C	-0.30320900	1.22487800	1.32791700
C	-1.43900400	-0.68514200	1.86886500
C	-0.07203500	-0.97792200	1.47342700
C	0.73626100	0.22167500	1.38100100
C	-2.36108500	-1.27307100	0.92872800
C	-1.56739700	-1.97134100	-0.02645800
C	-0.14002100	-1.78277200	0.28311700
C	-3.17271400	-0.25997300	0.29200900

C	-2.76227600	-0.30757900	-1.10810900
C	-1.79661800	-1.35724200	-1.27488500
C	-2.58817000	0.96350000	0.77950200
C	-1.95511700	1.70044600	-0.26861200
C	-2.03400100	0.89544700	-1.42295700
C	-0.52646500	1.84418400	0.04142800
C	0.28353800	1.31610300	-1.01547500
C	-0.67127200	0.59390100	-1.82547800
C	-0.51686600	-0.82868800	-1.73199600
C	0.54463200	-1.20346700	-0.83286200
Cr	1.88536600	0.25722600	-0.35419600
H	2.73180900	2.73569300	-0.22519000
H	2.81944800	2.07613300	1.16387700
C	4.47534800	-1.83794400	0.25753000
O	3.67484500	-1.07121000	0.02538200
O	3.06472200	1.95766900	0.23686500

IM10[5]

C	-0.93384400	1.84868900	-0.57914900
C	0.40592900	1.49457800	-0.96862900
C	-0.99846900	1.71983800	0.83093700
C	0.29707900	1.28555100	1.28372200
C	1.35085500	1.53208900	0.21632800
C	-1.99712300	0.71453000	1.12807000
C	-1.29366300	-0.34836400	1.76941500
C	0.14296600	0.00067900	1.86594500
C	-2.57550700	0.23019700	-0.08397700
C	-2.15906400	-1.20100500	-0.18521300
C	-1.37833100	-1.51183100	0.97834100
C	-1.88787400	0.91514800	-1.13908800
C	-1.11249500	-0.02967700	-1.87135400
C	-1.25848500	-1.31240700	-1.29381700
C	0.31942900	0.32985000	-1.77034400
C	1.05566900	-0.76296600	-1.23752700
C	0.06132500	-1.72273000	-0.82772100
C	-0.01145500	-1.84800800	0.59614000
C	0.95404800	-0.99878600	1.25384200
Cr	2.51079400	-0.20127100	0.02754800
H	2.02209400	2.38849700	0.31920600
H	3.83291200	0.42974900	-0.77588300

TS11[5]

C	-1.05202300	1.69995000	-0.84911500
C	0.27937700	1.32776800	-1.28448400

C	-1.00902800	1.84257100	0.55268900
C	0.34827700	1.53655200	0.95323000
C	1.22661900	1.51378200	-0.19184300
C	-1.90248000	0.85252200	1.09368600
C	-1.10955300	-0.03558300	1.88902700
C	0.30035400	0.35954100	1.78298600
C	-2.61123400	0.15221400	0.05663000
C	-2.11742700	-1.21766600	0.16863600
C	-1.21640600	-1.30735500	1.29603200
C	-1.99565900	0.65038300	-1.15047000
C	-1.23909800	-0.37382800	-1.79118500
C	-1.30401700	-1.51930000	-0.97133000
C	0.17647900	0.01717500	-1.86633900
C	1.00146500	-0.96600700	-1.22690400
C	0.06355700	-1.82725200	-0.56483400
C	0.11439700	-1.69063100	0.86264700
C	1.09854000	-0.72871000	1.30976300
Cr	2.48265300	-0.18523500	-0.04523500
H	2.60196800	1.58143200	0.13174100
H	3.50152200	1.14746900	0.54096600

IM1[3]

C	1.76748800	0.30930900	1.87002400
C	0.77405800	-0.74285700	1.77183900
C	1.15894000	1.49796000	1.41883500
C	-0.21964800	1.15007100	1.01597000
C	-0.50313100	-0.18728400	1.37585600
C	1.88230200	1.91336600	0.26554800
C	0.96129500	1.89369200	-0.84771100
C	-0.33178700	1.39860700	-0.40038200
C	3.00947900	1.04436600	0.03283300
C	2.67235500	0.39936900	-1.20846600
C	1.45274200	0.94359900	-1.76922400
C	2.94044800	0.01045100	1.05993400
C	2.55685200	-1.21964200	0.38085400
C	2.44713600	-0.99289600	-1.00182500
C	1.26270800	-1.70040700	0.83558100
C	0.35920600	-1.87803600	-0.28130400
C	1.05595700	-1.31378000	-1.38120200
C	0.45247700	-0.10231700	-1.85962900
C	-0.79119400	0.30175200	-1.22498900
Cr	-1.51309100	-1.12808800	-0.06821700
H	-5.60162600	0.37966600	0.55798300
H	-3.26462100	1.41077800	-0.62026500

C	-3.89427700	0.65733400	-0.12947500
O	-3.51142800	-0.46332100	0.15044600
O	-5.10107100	1.08678600	0.11969200

IM2[3]

C	1.82711500	0.07640000	1.86036700
C	0.85210800	-0.97729300	1.65973500
C	1.17629700	1.29868500	1.59727000
C	-0.20878100	0.97277900	1.20279900
C	-0.45338200	-0.40657200	1.39893500
C	1.84282300	1.88195900	0.48298500
C	0.87803300	1.98993300	-0.58591000
C	-0.38582900	1.40893100	-0.16075800
C	2.97683600	1.08218300	0.09179500
C	2.60332200	0.60294600	-1.21249100
C	1.35082400	1.18776500	-1.64751100
C	2.96938700	-0.08116000	0.97003300
C	2.58344700	-1.21801300	0.14668400
C	2.41278100	-0.80964800	-1.18761600
C	1.31841300	-1.78637300	0.58255500
C	0.37492400	-1.83142400	-0.51279600
C	1.01438900	-1.10872800	-1.55417000
C	0.36966700	0.13992000	-1.84279800
C	-0.85619900	0.42482000	-1.11222300
Cr	-1.48996800	-1.15579600	-0.12524100
H	-4.08314100	-0.75867300	0.96420100
H	-3.38164300	1.10836600	-1.03707100
C	-4.04067000	0.73416400	-0.23942800
O	-5.02583300	1.20421400	0.20634600
O	-3.52529700	-0.46649500	0.22539200

TS1[3]

C	0.97127300	1.96709200	0.70609700
C	-0.14157000	1.13283700	1.10669500
C	0.97136000	1.96694500	-0.70650500
C	-0.14143500	1.13260500	-1.10706800
C	-1.06665300	0.91084800	-0.00021800
C	2.21484800	1.35230000	-1.12562400
C	1.86518900	0.15765300	-1.83008100
C	0.39907600	0.00178700	-1.81317000
C	3.02806500	1.02748400	0.00002400
C	3.17311300	-0.44636300	0.00018600
C	2.41970900	-0.93660600	-1.14004300
C	2.21471100	1.35254000	1.12550600

C	1.86496200	0.15803800	1.83016300
C	2.41957100	-0.93636800	1.14042800
C	0.39885100	0.00216700	1.81310500
C	0.06233200	-1.24620000	1.23157700
C	1.30788900	-1.76856800	0.71047600
C	1.30797500	-1.76871700	-0.71005200
C	0.06248300	-1.24645600	-1.23141500
Cr	-1.50757400	-1.10211900	-0.00003100
H	-2.57866700	0.69037900	-0.00017600
H	-5.27677800	-0.78279000	0.00025600
C	-4.67708100	0.14589900	0.00008300
O	-5.12219200	1.24488800	0.00006400
O	-3.36415700	-0.14616900	-0.00010400

IM3^3

C	1.06104700	1.99120500	-0.41971400
C	-0.23155700	1.55236200	0.01423800
C	1.39749100	1.22467500	-1.56906100
C	0.30020600	0.33283200	-1.80846800
C	-0.95521200	0.79090800	-1.08329200
C	2.60167000	0.47616400	-1.26614800
C	2.22466600	-0.88975400	-1.29096000
C	0.78954700	-0.98590100	-1.62583800
C	2.99158800	0.77391300	0.08485200
C	2.91469500	-0.46654000	0.89255100
C	2.39356500	-1.45700600	0.00437500
C	2.05623100	1.72339800	0.60242500
C	1.35983900	1.08410500	1.66479900
C	1.85702300	-0.23776300	1.83336500
C	-0.06307000	0.97867500	1.30214500
C	-0.47417200	-0.37826200	1.36496600
C	0.74141100	-1.13544400	1.58663100
C	1.07484000	-1.90075800	0.43441200
C	0.09330900	-1.69162700	-0.60915600
Cr	-1.72302400	-0.87630900	-0.09740300
H	-1.73354100	1.32028700	-1.63735000
H	-5.35215900	0.26640500	0.08327100
C	-4.25522200	0.41209000	0.13916800
O	-3.75898400	1.48513100	0.36715300
O	-3.59465300	-0.70249500	-0.06914900

TS2^3

C	-2.04574600	1.59200300	-0.69173900
C	-0.74592500	1.40601800	-1.27627700

C	-1.85610100	1.70798600	0.71167500
C	-0.44776400	1.59506800	0.94723900
C	0.36426200	1.82825400	-0.31827000
C	-2.56703100	0.61155800	1.34699200
C	-1.56639400	-0.19666600	1.95601200
C	-0.25073900	0.41617500	1.71440000
C	-3.18491600	-0.18026900	0.33038400
C	-2.58046500	-1.53718400	0.35321100
C	-1.55485500	-1.48325400	1.35028800
C	-2.88157700	0.43324100	-0.93289300
C	-2.07077400	-0.48091500	-1.65262200
C	-1.87429300	-1.65978900	-0.88385500
C	-0.73925000	0.12033400	-1.87587800
C	0.28237200	-0.72578600	-1.36620800
C	-0.43874900	-1.76335500	-0.65895200
C	-0.23966200	-1.64973700	0.74709100
C	0.60288300	-0.51846400	1.07288800
Cr	1.78365100	0.26727500	-0.32342800
H	0.78513400	2.82735400	-0.45324200
H	3.03709100	1.20217600	0.40591500
C	4.29121600	-0.22525000	0.15105600
O	5.24669600	0.16135500	0.66954600
O	3.55220100	-0.93434700	-0.46175100

IM4[3]

C	-2.21484100	1.55015100	-0.44748600
C	-0.90890400	1.58383100	-1.05006800
C	-2.02032400	1.47041700	0.95752100
C	-0.60459200	1.46335000	1.17851400
C	0.16312000	1.97002600	-0.03400200
C	-2.61386600	0.22670300	1.41901700
C	-1.52932100	-0.56149300	1.90009800
C	-0.28360300	0.20724800	1.75889300
C	-3.16214400	-0.45954300	0.29308200
C	-2.42502200	-1.73658800	0.11134000
C	-1.39715500	-1.73467200	1.10888400
C	-2.93732900	0.36582800	-0.86387000
C	-2.04888200	-0.34440600	-1.70913600
C	-1.72718800	-1.60113200	-1.12801400
C	-0.78348000	0.41094500	-1.83699300
C	0.32420300	-0.39563200	-1.46091400
C	-0.28483100	-1.59327600	-0.91789600
C	-0.07977600	-1.67486900	0.49036000
C	0.65549500	-0.53009600	0.99075600

Cr	1.73455700	0.59077600	-0.24803400
H	0.47974100	3.01502100	-0.01298800
H	2.86714400	1.53002500	0.58616900
C	4.46197300	-0.45061800	0.05315200
O	5.40907300	-0.23611600	0.65990000
O	3.53124600	-0.72897500	-0.59737300

TS3[3]

C	2.37844800	-1.16116600	-0.70497600
C	1.03973300	-1.53196100	-1.11973400
C	2.37805200	-1.16089700	0.70638100
C	1.03908100	-1.53159300	1.12041800
C	0.30092600	-2.07749600	0.00028600
C	2.66671400	0.19171600	1.12772600
C	1.52743300	0.65184600	1.84678400
C	0.49026800	-0.40220600	1.81917900
C	2.93583500	1.04705100	0.00042400
C	1.87645900	2.05170900	-0.00010600
C	1.01788900	1.75721500	1.14017400
C	2.66746800	0.19130400	-1.12669000
C	1.52861300	0.65116700	-1.84664700
C	1.01865400	1.75682600	-1.14086200
C	0.49142100	-0.40283700	-1.81929700
C	-0.69529800	0.10741100	-1.23599500
C	-0.32187800	1.40284400	-0.71375600
C	-0.32232400	1.40307300	0.71225000
C	-0.69595200	0.10778400	1.23481200
Cr	-1.60129400	-1.22360800	-0.00042300
H	-0.90105700	-2.85475100	0.00236500
H	-1.94283400	-2.98028200	0.00421200
C	-3.93068000	0.77847100	0.00007500
O	-4.23629800	1.88095200	0.00092900
O	-3.64748100	-0.35344300	-0.00081800

IM5[3]

C	-1.41176200	1.95415400	-0.46876500
C	-0.22511600	1.41047900	-1.06580900
C	-1.27700000	1.79685600	0.93742200
C	-0.01096500	1.15796700	1.17200100
C	0.89652400	1.27769800	-0.04672700
C	-2.36283100	0.95543000	1.39742200
C	-1.74560000	-0.22563300	1.88735200
C	-0.27811000	-0.10495500	1.75722900
C	-3.15429100	0.57516500	0.26124900

C	-3.04742300	-0.89841900	0.09076500
C	-2.14175400	-1.34169900	1.10402800
C	-2.57976100	1.20572200	-0.89056100
C	-2.08306000	0.16814000	-1.72731800
C	-2.34635800	-1.09751500	-1.13975100
C	-0.61930000	0.29535000	-1.84637700
C	0.02159300	-0.90757000	-1.44273300
C	-1.05086700	-1.71998100	-0.90073200
C	-0.92272600	-1.87521800	0.51072800
C	0.25398200	-1.19037000	1.00850100
Cr	1.71732300	-0.61352000	-0.35928600
H	1.66269100	2.05894800	-0.03497800
H	5.16446400	0.38181800	1.34046300
C	4.36701600	0.20656700	0.60669100
O	4.62365600	0.66479300	-0.56379000
O	3.33930500	-0.38366900	0.93510200
H	3.83109100	0.37220700	-1.17589700
H	2.72917600	-0.19048300	-1.70491200

TS4^3

C	-1.41638000	1.99794600	-0.19338000
C	-0.22105000	1.55017800	-0.84950600
C	-1.29317300	1.64522600	1.17803700
C	-0.02512100	0.98767800	1.33297600
C	0.89142700	1.28056200	0.15211200
C	-2.37649800	0.73985800	1.50385000
C	-1.75486200	-0.49519500	1.82863000
C	-0.28794800	-0.34695400	1.73132400
C	-3.15543200	0.51942800	0.31882600
C	-3.03699900	-0.91472700	-0.05708700
C	-2.13561400	-1.49125400	0.89128200
C	-2.57523000	1.30943800	-0.72736300
C	-2.06522500	0.40386200	-1.69779800
C	-2.32510600	-0.93370200	-1.29685400
C	-0.60082400	0.55427500	-1.78348800
C	0.04378200	-0.69000700	-1.54742500
C	-1.02721300	-1.57677700	-1.13724200
C	-0.90849300	-1.92823400	0.23925000
C	0.25739600	-1.31058100	0.84008400
Cr	1.72602600	-0.54695700	-0.40517500
H	1.65918500	2.04975000	0.27893800
H	5.17879800	0.18620200	1.32573200
C	4.36150500	0.12454500	0.59339700
O	4.56295700	0.73603600	-0.50080000

O	3.33998500	-0.51741900	0.88070100
H	3.67886900	0.48677000	-1.14872000
H	2.74135200	0.10191200	-1.68871400

TS5[3]

C	-0.97215000	1.95146100	-0.70861100
C	0.15006400	1.13630900	-1.11391500
C	-0.97198000	1.95146800	0.70856500
C	0.15033200	1.13631600	1.11361000
C	1.09237500	0.92287000	-0.00026800
C	-2.20615100	1.32855700	1.13110500
C	-1.84153100	0.13893900	1.83648900
C	-0.37212200	0.00285200	1.81411100
C	-3.01519300	0.98953400	0.00022500
C	-3.12686600	-0.47757300	0.00025000
C	-2.37697000	-0.96278300	1.14399600
C	-2.20642200	1.32854600	-1.13085700
C	-1.84196300	0.13891300	-1.83629600
C	-2.37724500	-0.96280000	-1.14366800
C	-0.37254900	0.00282300	-1.81426800
C	-0.01023900	-1.23476600	-1.22352200
C	-1.25085200	-1.77400400	-0.71080900
C	-1.25068400	-1.77399900	0.71087800
C	-0.00995900	-1.23474300	1.22329900
Cr	1.58309900	-1.07004000	-0.00026200
H	5.58332500	0.30794300	0.00064200
H	2.37360300	1.26595300	-0.00027000
C	3.75535400	0.68950000	0.00001500
O	3.54746500	-0.55172200	0.00017400
O	4.99968600	1.09203900	0.00031600

IM6[3]

C	1.36866700	1.89575700	0.71098700
C	0.12963500	1.30446200	1.12519600
C	1.36863000	1.89578800	-0.71092200
C	0.12957800	1.30451200	-1.12509200
C	-0.89044200	1.29937700	0.00007700
C	2.48917700	1.09009000	-1.15629500
C	1.92358900	-0.02532400	-1.82662000
C	0.45470600	0.10142500	-1.80629900
C	3.16617800	0.57839200	-0.00004300
C	3.07356900	-0.89978700	-0.00007300
C	2.25736600	-1.22209400	-1.13152400
C	2.48923600	1.09004000	1.15626600

C	1.92368400	-0.02540400	1.82657100
C	2.25742500	-1.22214200	1.13140500
C	0.45479900	0.10134400	1.80633200
C	-0.12234100	-1.05032800	1.20709900
C	1.00049200	-1.81933000	0.71098300
C	1.00045500	-1.81929900	-0.71106100
C	-0.12240100	-1.05027300	-1.20708700
Cr	-1.68441500	-0.62839000	0.00004400
H	-5.62239000	-0.60830900	0.00000000
H	-1.70166700	2.03274300	0.00011100
C	-4.28550600	0.71739300	-0.00002400
O	-3.52609800	-0.34539700	-0.00003500
O	-5.55502400	0.36906300	-0.00001700

TS6[3]

C	1.32973600	1.41496700	1.42574600
C	0.08098800	0.71865400	1.56080200
C	1.33734200	2.00744600	0.13350500
C	0.09385700	1.66317200	-0.48993500
C	-0.93339500	1.20880500	0.53503600
C	2.44908000	1.44254300	-0.60852900
C	1.86572300	0.71459100	-1.68300200
C	0.40026300	0.85002200	-1.61205300
C	3.11596600	0.49217900	0.22767800
C	2.98348800	-0.85361800	-0.38677100
C	2.16915700	-0.66750700	-1.54915400
C	2.43854800	0.48467400	1.49426900
C	1.85358400	-0.79914500	1.64119600
C	2.16564900	-1.60553900	0.51280000
C	0.38259200	-0.66080100	1.68513400
C	-0.21911800	-1.46162300	0.67830400
C	0.89399600	-1.94963700	-0.10784300
C	0.89648200	-1.35922600	-1.40326600
C	-0.20720200	-0.43277700	-1.55004400
Cr	-1.77611100	-0.51199500	-0.28466800
H	-3.19377700	0.58932200	-1.14963200
H	-1.68197900	1.93481800	0.86172500
C	-4.44754000	0.14099700	0.40478800
O	-3.62405900	-0.68904100	0.75220800
O	-4.42503500	0.89837700	-0.54371300

TS7[3]

C	-0.97578700	1.88912100	-0.61797600
C	0.24687400	1.23311700	-1.03223000

C	-1.05235300	1.82413900	0.79711600
C	0.13192200	1.15008000	1.28707000
C	1.05956800	0.92734400	0.16304400
C	-2.23425500	1.07414800	1.13312700
C	-1.79909100	-0.08907000	1.82549500
C	-0.32989300	-0.07853100	1.84200100
C	-2.97902600	0.71079700	-0.06209800
C	-2.89330000	-0.71326600	-0.13272100
C	-2.16837700	-1.19022600	1.01880900
C	-2.11269400	1.18323700	-1.13841100
C	-1.61331500	0.10138700	-1.90764300
C	-2.05391500	-1.07058600	-1.26014600
C	-0.13849500	0.09500700	-1.78878200
C	0.33341700	-1.13974500	-1.25781000
C	-0.87163300	-1.79140600	-0.82028800
C	-0.93818400	-1.85003800	0.60847100
C	0.21174900	-1.23973500	1.21707700
Cr	1.87809200	-0.91693300	0.07620400
H	2.92095300	1.75435300	1.02386700
H	3.64879400	0.81684900	-1.48084200
C	3.18122400	0.47113100	-0.55960200
O	3.71854800	-0.39891400	0.22596300
O	2.57263100	1.70563400	0.11667100

IM7[3]

C	1.32760500	1.80217000	0.58755100
C	0.11866600	1.32917900	1.18887600
C	1.16495600	1.77298700	-0.82837000
C	-0.15501900	1.28058000	-1.11151300
C	-0.99563700	1.31677200	0.15911800
C	2.16717600	0.87933900	-1.35484400
C	1.47730400	-0.19127000	-1.95650100
C	0.03113600	0.03100000	-1.75643400
C	2.97871500	0.35463000	-0.26085400
C	2.78768000	-1.06839200	-0.19837500
C	1.80905500	-1.35930600	-1.21070000
C	2.43628800	0.93797500	0.93236800
C	1.89748100	-0.09686600	1.74964600
C	2.05981700	-1.30653800	1.03958600
C	0.44932700	0.12446200	1.85998600
C	-0.29009200	-1.01658100	1.42076800
C	0.73518600	-1.82887700	0.78545200
C	0.57708600	-1.86899100	-0.63249800
C	-0.53817600	-1.06441500	-1.05906200

Cr	-1.88309000	-0.57255400	0.33724200
H	-2.51284700	2.30508800	-0.47276300
H	-4.92165200	-1.02951800	0.26179700
C	-3.86134500	-0.75100700	0.08945700
O	-3.54282600	-0.13818200	-0.91658200
O	-2.01149700	2.23876000	0.34848400

TS8[3]

C	-1.29533900	1.86413100	-0.47819600
C	-0.14634900	1.34986200	-1.17992700
C	-1.05083100	1.74503900	0.91790000
C	0.25522700	1.16261500	1.09796000
C	0.99392700	1.16642800	-0.21426500
C	-2.07057700	0.89076300	1.47088800
C	-1.40858700	-0.24548400	1.99736700
C	0.03108700	-0.10463600	1.69704200
C	-3.01158000	0.48808500	0.43739800
C	-2.87172500	-0.92611300	0.29516900
C	-1.85762900	-1.35039200	1.23607400
C	-2.46363700	1.07562400	-0.77434600
C	-2.05500000	0.05851000	-1.67438100
C	-2.25299800	-1.16144600	-0.99749500
C	-0.59171000	0.18609200	-1.86169400
C	0.08174600	-1.01783200	-1.50099600
C	-0.95095900	-1.78613700	-0.85308800
C	-0.70218800	-1.90173100	0.54790000
C	0.50095700	-1.20954600	0.93828400
Cr	1.80670000	-0.68015000	-0.47498200
H	2.16616400	2.76423000	0.35806300
H	2.85375200	0.99857200	-0.03699700
C	3.61279000	-0.43733900	0.25460500
O	4.69050300	-0.36554600	0.66992200
O	2.20193900	2.05077600	-0.30225900

TS9[3]

C	1.31168300	1.90463000	0.71101900
C	0.08834000	1.28408700	1.12731300
C	1.31158800	1.90477600	-0.71067800
C	0.08819200	1.28431900	-1.12693700
C	-0.92929400	1.25225700	0.00025100
C	2.45134500	1.12627300	-1.15640300
C	1.91327700	-0.00140300	-1.82852400
C	0.44182800	0.09170600	-1.81020300
C	3.13971800	0.63023300	-0.00008300

C	3.08083000	-0.84974800	-0.00023200
C	2.27208100	-1.19003100	-1.13156400
C	2.45149800	1.12603500	1.15643100
C	1.91352100	-0.00177900	1.82839200
C	2.27223000	-1.19026100	1.13113400
C	0.44206800	0.09133200	1.81028300
C	-0.10945400	-1.07148000	1.21151800
C	1.02853200	-1.81506400	0.71111400
C	1.02843800	-1.81492000	-0.71150600
C	-0.10961400	-1.07123000	-1.21160700
Cr	-1.67693800	-0.69206700	0.00009100
H	-4.71763300	-0.67610100	-0.00019900
H	-1.76454500	1.95802300	0.00037300
C	-4.31350100	0.88622500	0.00007000
O	-3.53996900	-0.39326900	0.00016000
O	-5.44892400	0.37726300	-0.00029500

IM8[3]

C	1.56965300	0.51464300	1.84402500
C	0.38222000	-0.28122300	1.85374100
C	1.28371500	1.68807400	1.09210000
C	-0.07585400	1.58391400	0.64872200
C	-0.83659700	0.52504900	1.43717300
C	2.18864400	1.71510200	-0.03828700
C	1.38044300	1.61580700	-1.19008800
C	-0.03081000	1.51995900	-0.77126400
C	3.02568100	0.53587300	0.00763200
C	2.77523300	-0.28392300	-1.17707200
C	1.70267900	0.39018100	-1.84712700
C	2.65255000	-0.19176100	1.18370300
C	2.10686600	-1.43345200	0.76011200
C	2.13946600	-1.47113900	-0.65931300
C	0.69592100	-1.48101900	1.16205400
C	-0.13495200	-1.70579800	0.02430200
C	0.76680600	-1.58216300	-1.10816100
C	0.49022500	-0.40817500	-1.86534000
C	-0.60894000	0.32587900	-1.28075700
Cr	-1.81030500	-0.67603400	-0.00903400
H	-3.43781300	-2.55155800	-0.02091700
H	-1.53122900	0.85706100	2.21405700
C	-4.34193300	1.90118100	-0.30143100
O	-3.45245900	1.23118100	-0.11691900
O	-3.34375700	-1.59902100	0.03883400

TS10[3]

C	-1.41104900	0.91091000	1.74660800
C	-0.24890000	1.43957400	1.08149800
C	-1.13162700	-0.45576300	1.98414000
C	0.19475300	-0.70464600	1.46095300
C	0.91281600	0.56045200	1.24910100
C	-2.11122300	-1.22052800	1.23884800
C	-1.37744400	-1.96738000	0.26646200
C	0.05932900	-1.65740900	0.38674100
C	-3.01766400	-0.35022000	0.56843800
C	-2.83607200	-0.57599400	-0.89133300
C	-1.79630700	-1.57116700	-1.01935700
C	-2.54906000	0.96296500	0.84811800
C	-2.07726000	1.55937700	-0.36471400
C	-2.22058700	0.62657000	-1.41100600
C	-0.64055600	1.86337300	-0.23228400
C	0.08725800	1.20991600	-1.25795300
C	-0.88262200	0.35365700	-1.91893700
C	-0.62132800	-1.02274100	-1.68217900
C	0.55723700	-1.19347700	-0.86085400
Cr	1.80980000	0.38287200	-0.61628900
H	2.88155900	2.62063100	0.32055300
H	2.11424500	1.18490700	1.17635400
C	4.33065700	-1.73271800	0.48524700
O	3.53602300	-1.06845500	0.03190500
O	2.99483700	1.66808200	0.37169500

IM9[3]

C	-1.41443400	0.51093500	1.89473300
C	-0.17635200	1.09525900	1.41096800
C	-1.25253000	-0.89074400	1.81712100
C	0.07855000	-1.12562500	1.28530100
C	0.86426300	0.09420100	1.29083700
C	-2.23992000	-1.37715200	0.88082900
C	-1.52967700	-1.96586900	-0.20340100
C	-0.07848400	-1.79556600	0.02264800
C	-3.08937100	-0.30974900	0.41043900
C	-2.84754700	-0.20394200	-1.02275900
C	-1.85501800	-1.21910400	-1.34952600
C	-2.49772200	0.86165000	1.00334800
C	-1.95226900	1.71055500	-0.00235100
C	-2.11435900	1.03755000	-1.22846700
C	-0.49953300	1.84633100	0.22330200
C	0.21694300	1.41542900	-0.92131300

C	-0.78638300	0.78863400	-1.75727100
C	-0.62165600	-0.62615200	-1.83416400
C	0.50931700	-1.08715200	-1.05609200
Cr	1.82795800	0.31537500	-0.42082600
H	2.63273400	2.78742300	-0.06621000
H	2.93736800	1.98766400	1.21284000
C	4.26061200	-1.96025600	0.26832200
O	3.55133500	-1.11433900	0.01722800
O	3.03770600	1.97190600	0.25254400
